# MED12 mutation activates the tryptophan/kynurenine/AHR pathway to promote growth of uterine leiomyomas

**DOI:** 10.1172/jci.insight.171305

**Published:** 2023-09-22

**Authors:** Azna Zuberi, Yongchao Huang, Ariel J. Dotts, Helen Wei, John S. Coon, Shimeng Liu, Takashi Iizuka, Olivia Wu, Olivia Sotos, Priyanka Saini, Debabrata Chakravarti, Thomas G. Boyer, Yang Dai, Serdar E. Bulun, Ping Yin

**Affiliations:** 1 Division of Reproductive Science in Medicine, Department of Obstetrics & Gynecology, Feinberg School of Medicine, Northwestern University, Chicago, Illinois, USA.; 2 Department of Biomedical Engineering, University of Illinois at Chicago, Chicago, Illinois, USA.; 3 Department of Molecular Medicine, Institute of Biotechnology, University of Texas Health Science Center at San Antonio, San Antonio, Texas, USA.

**Keywords:** Metabolism, Reproductive Biology, Amino acid metabolism, Expression profiling, Fibrosis

## Abstract

Uterine leiomyomas cause heavy menstrual bleeding, anemia, and pregnancy loss in millions of women worldwide. Driver mutations in the transcriptional mediator complex subunit 12 (MED12) gene in uterine myometrial cells initiate 70% of leiomyomas that grow in a progesterone-dependent manner. We showed a distinct chromatin occupancy landscape of MED12 in mutant MED12 (mut-MED12) versus WT-MED12 leiomyomas. Integration of cistromic and transcriptomics data identified tryptophan 2,3-dioxygenase (TDO2) as the top mut-MED12 target gene that was significantly upregulated in mut-MED12 leiomyomas when compared with adjacent myometrium and WT-MED12 leiomyomas. TDO2 catalyzes the conversion of tryptophan to kynurenine, an aryl hydrocarbon receptor (AHR) ligand that we confirmed to be significantly elevated in mut-MED12 leiomyomas. Treatment of primary mut-MED12 leiomyoma cells with tryptophan or kynurenine stimulated AHR nuclear translocation, increased proliferation, inhibited apoptosis, and induced AHR-target gene expression, whereas blocking the TDO2/kynurenine/AHR pathway by siRNA or pharmacological treatment abolished these effects. Progesterone receptors regulated the expression of AHR and its target genes. In vivo, TDO2 expression positively correlated with the expression of genes crucial for leiomyoma growth. In summary, activation of the TDO2/kynurenine/AHR pathway selectively in mut-MED12 leiomyomas promoted tumor growth and may inform the future development of targeted treatments and precision medicine.

## Graphical abstract

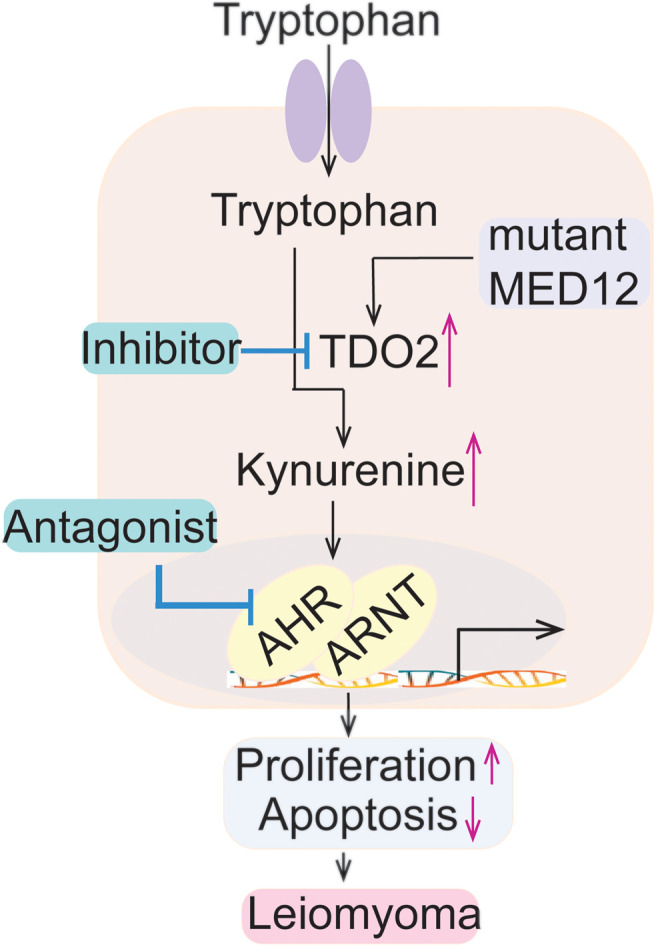

## Introduction

Uterine leiomyomas (LM, fibroids) represent the most common benign tumors in women. By age 50 years, up to 80% of all women may be diagnosed with at least 1 LM, and 15%–30% will develop severe symptoms (1, 2). These smooth muscle tumors disrupt uterine function and cause excessive uterine bleeding, anemia, labor obstruction, and urinary incontinence (3–6). African American women develop larger LM at a higher rate and at earlier ages than European American women and develop more severe symptoms (7–10). To remove or destroy LM, approximately 250,000 hysterectomies or myomectomies, and many less-invasive procedures with morbid affects, are performed in the United States annually, costing up to $34.4 billion (11). Gonadotropin-releasing hormone (GnRH) analogues and antagonists, the only class of pharmaceuticals approved in the United States for treatment of LM since the 1990s, target the production of ovarian hormones but cause significant side effects and are not suitable for long-term use (12, 13). There is, thus, an urgent need for new medical options to treat LM.

High-throughput sequencing analysis uncovered different gene mutations in LM, including mediator complex subunit 12 (MED12) gene mutation (mut-MED12), high mobility group AT-hook 2 (HMGA2) translocations, biallelic loss of fumarate hydratase (FH), collagen (COL4A5–COL4A6) gene deletions, and germline mutations in the SRCAP members YEATS4 and ZNHIT1 (14, 15), with mut-MED12 contributing to approximately 70% of all LM (16). The same MED12 mutations were found in 70% of breast fibroadenomas, another ovarian steroid-dependent tumor (17). Knockin of G44D mutant MED12, the most frequent mutation subtype, in mouse uterine tissue gives rise to a LM-like phenotype (18), suggesting that MED12 mutation is a driver of LM tumorigenesis.

MED12, along with MED13, cyclin C (CycC), and cyclin-dependent kinase 8 (CDK8), constitutes the 4-subunit mediator kinase submodule, which dynamically interacts with a 26-subunit core mediator (19, 20). The mediator complex modulates gene transcriptional activity by bridging regulatory signals from DNA-bound transcription factors directly to RNA polymerase II (20). In general, individual mediator subunits interact specifically with their corresponding transcription factors, and deletion of these subunits often affects the expression of primary target genes and pathways controlled by their cognate transcription factors (21). The human mediator complex was reported to interact with several nuclear receptors (22–24). For example, estrogen receptor 1 (ESR1) interacts with the MED1 subunit, which is required for ESR1-mediated transcription and estradiol/ESR1-dependent mammary stem cell function and breast cancer cell growth (25–28). The specific transcription factors interacting with MED12 in LM remain unknown. Studies have demonstrated that uterine LM–linked mut-MED12 destabilizes the CDK8 activation loop and decreases mediator-associated CDK activity (29–33). LM-carrying mut-MED12 show distinct DNA methylation (34, 35), histone modification (15, 36–38), and gene expression patterns (39). However, the contribution of signaling pathways affected by mut-MED12 in LM growth remains uncharacterized.

In this study, we mapped genome-wide chromatin occupancy of MED12 in LM tissues expressing mut- or WT-MED12 and their matched myometrium (MyoF) tissues to explore whether mut-MED12 alters the interaction profile of MED12 with chromatin. Integrative analysis of ChIP-Seq and RNA-Seq data identified tryptophan (Trp) 2,3-dioxygenase (TDO2) as a MED12 target gene that was aberrantly upregulated in mut-MED12 LM compared with WT-MED12 LM, consistent with recent reports (40, 41). TDO2 catalyzes the first and rate-limiting step of Trp breakdown to kynurenine (Kyn), which functions as an endogenous ligand for the aryl hydrocarbon receptor (AHR) (42–46). AHR is a ligand-activated transcription factor known to mediate the toxic and carcinogenic effects of a wide variety of environmental contaminants including 2,3,7,8-tetrachlorodibenzo-p-dioxin (TCDD or dioxin) (47–49). Studies have demonstrated a critical role of TDO2/Kyn/AHR pathway activation in the promotion of tumor growth (42, 50–55). Here, we investigated the role of this pathway in LM growth.

## Results

### RNA-Seq analysis identifies mRNA expression differences between WT- and mut-MED12 LM and adjacent MyoF.

**Figure 1:**
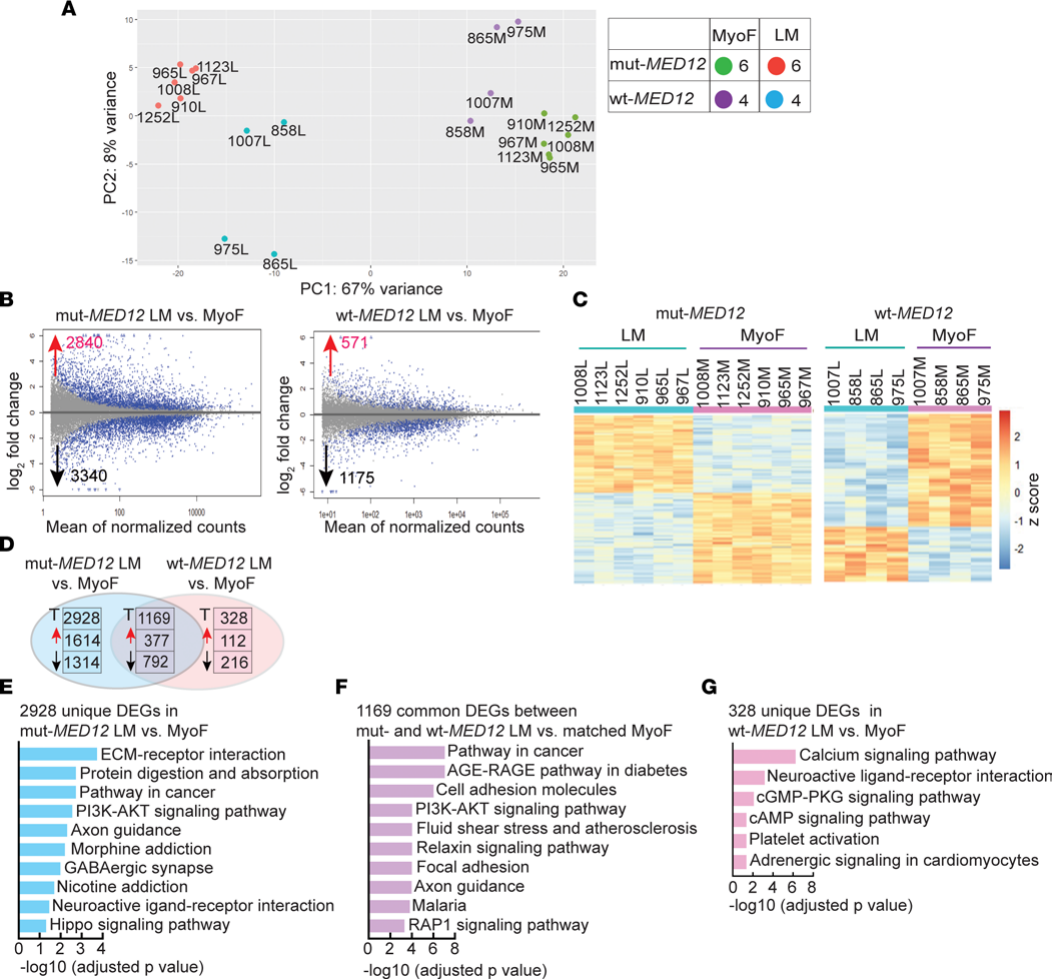
RNA-Seq analysis identifies mRNA expression differences between WT- and mut-*MED12* LM and adjacent MyoF. (**A**) Gene expression in G44D mut-MED12 LM (*n* = 6), WT-MED12 LM (*n* = 4), and their matched MyoF (*n* = 10) tissues was examined by unsupervised PCA. L, LM; M, MyoF. (**B** and **C**) MA (log_2_ fold change and means average) plots and heatmap showing differences in gene expression (up- and downregulated) in LM versus matched MyoF tissues. Differential gene expression was determined using a cutoff of *P*_adj_ < 0.05. Gene expression levels relative to the mean expression are shown as row *Z* scores. (**D**) Venn diagrams showing unique or shared differentially expressed genes (DEGs) between mut- and WT-MED12 LM versus their matched MyoF (fold change >1.5, *P*_adj_ < 0.05). T, total. (**E**–**G**) Top 10 enriched KEGG pathways in DEGs uniquely present in mut-MED12 LM versus matched MyoF (**E**) and in DEGs shared between mut- and WT-MED12 LM versus their matched MyoF (**F**), and 6 enriched pathways in DEGs uniquely present in WT-MED12 LM versus matched MyoF (**G**).

The genotypes and clinical and biological characteristics of human tissue samples used in this study are listed in Supplemental Table 1 (supplemental material available online with this article; https://doi.org/10.1172/jci.insight.171305DS1). We performed RNA-Seq using RNA isolated from WT-MED12 LM that do not carry MED12 mutation (*n* = 4), G44D mut-MED12 LM (*n* = 6), and adjacent MyoF biopsies (*n* = 10). To minimize genetic and therefore biological heterogeneity among the mut-MED12 LM tumors (56), we used only a specific mutation — i.e., G44D mut-MED12 LM — for RNA-Seq and ChIP-Seq. Unsupervised principal component analysis (PCA) showed that the samples were primarily grouped by disease state (MyoF versus LM) (Figure 1A). The transcriptomes of LM tissues were further clearly separated by mutation status (mut-MED12 versus WT-MED12).

A comparison between mut-MED12 LM and matched adjacent MyoF revealed 6,180 significantly differentially expressed genes (DEGs; adjusted *P* [*P*_adj_] < 0.05), with 2,840 genes upregulated and 3,340 downregulated in LM tissues (Figure 1, B and C). Among them, 2,682 genes exhibited a > 2-fold change in expression between MyoF and LM, while 1,033 genes were differentially expressed at > 4-fold. Recent RNA-Seq studies by other groups also report a large number of genes differentially expressed between mut-MED12 LM and MyoF. Using the cutoff of *P*_adj_ < 0.05, Leistico et al. reported 8,203 genes differentially expressed between mut-MED12 LM and MyoF (37). Using the cutoff of FDR < 0.01, Moyo et al. reported 5,831 DEGs between mut-MED12 LM and MyoF (36). However, between WT-MED12 LM and matched MyoF, there were only 1,746 DEGs with, 571 genes upregulated and 1,175 downregulated in LM (Figure 1, B and C). Fewer DEGs detected in WT-MED12 versus MyoF may be due to genetic heterogeneity since these LM likely express a variety of mutations reported in the literature (14, 15). The full results of differential expression analysis are shown in Supplemental Tables 2 and 3. Using the cutoff of 1.5-fold and an *P*_adj_ < 0.05, 2,928 DEGs were uniquely present in mut-MED12 LM, whereas 1,169 DEGs overlapped in mut-MED12 and WT-MED12 LM (in comparison with matched MyoF) (Figure 1D). The top 10 up- and downregulated genes (based on fold-change) under each category are shown in Table 1. DAVID functional enrichment analysis demonstrates that genes differentially expressed only in mut-MED12 LM were enriched in functional pathways involved in extracellular matrix–receptor (ECM-receptor) interactions, protein digestion and absorption, and oncogenic pathways, among others (Figure 1E and Supplemental Table 4) (57, 58). The genes differentially expressed in both WT- and mut-MED12 LM were enriched in pathways important for the AGE/RAGE pathway in diabetic complications, in the PI3K/AKT pathway, and in fluid shear stress and atherosclerosis, among other pathways (Figure 1F and Supplemental Table 5). Using the same criteria, genes involved in calcium signaling, cGMP-PKG signaling, and cAMP signaling were enriched in DEGs only present in WT-MED12 LM (Figure 1G and Supplemental Table 6).

We noticed that MyoF samples adjacent to mut-MED12 LM also clustered together, separated from MyoF samples adjacent to WT-MED12 LM (Figure 1A). There were 237 genes significantly upregulated and 301 downregulated in MyoF adjacent to mut-MED12 LM compared with MyoF adjacent to WT-MED12 LM (Supplemental Figure 1, A and B; *P*_adj_ < 0.05). The pathways enriched in these DEGs included those involved in normal myometrium function and associated with disease processes, such as relaxin signaling, oncogenic pathways, and estrogen signaling (Supplemental Figure 1C and Supplemental Table 7), suggesting that MyoF tissues adjacent to LM may also be abnormal at the molecular level. In summary, these results are consistent with previous studies and suggest that mut-MED12 dysregulates downstream gene expression and activates distinct cellular signaling pathways to stimulate LM growth (34–37, 39).

### mut-MED12 alters the chromatin occupancy signature of MED12 with a shift of its binding sites from promoter regions to intronic/intergenic regions in mut-MED12 LM.

**Figure 2:**
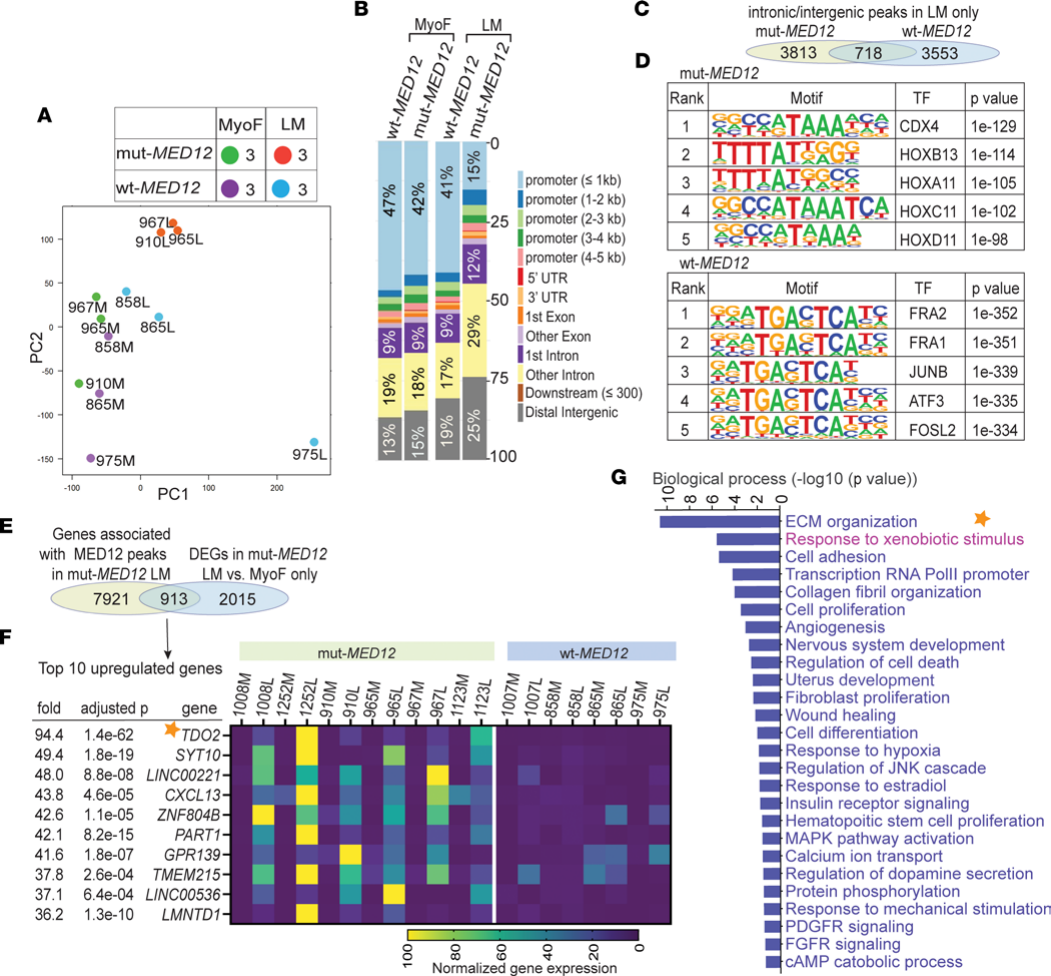
mut-MED12 alters chromatin occupancy signature of MED12 with a shift of its binding sites from promoter regions to intronic/intergenic regions in mut-*MED12* LM. (**A**) PCA plot showing the 2 most significant principal components. All binding sites identified as overlapping in replicate samples were merged and retained, and normalized read counts were computed at each site for each sample. (**B**) Stacked bar graphs showing percentage genomic distribution of unique MED12 peaks in mut-MED12 LM, WT-MED12 LM, and their matched MyoF. The legend indicates the genomic features. (**C**) Venn diagram showing shared and unique MED12-binding sites between mut- and WT-MED12 LM in intronic/intergenic regions. (**D**) The top 5 motifs enriched in 3,813 unique intronic/intergenic MED12 peaks in mut-MED12 LM and 3,553 unique intronic/intergenic MED12 peaks in WT-MED12 LM are indicated. TF, transcription factor. (**E**) Venn diagram showing DEGs only present in mut-MED12 LM versus MyoF and contained MED12 binding sites in mut-MED12 LM. (**F**) Heatmap showing percentage normalized gene expression of top 10 upregulated genes among the 913 genes shown in **E**. L, LM; M, MyoF. (**G**) GO biological processes (DAVID) of significantly enriched in the 913 genes shown in **E**.

To determine whether mut-MED12 alters the genome-wide MED12 and chromatin interaction landscape in LM, we performed MED12 ChIP-Seq on chromatin isolated directly from snap-frozen tissues of WT-MED12 LM (*n* = 3), G44D mut-MED12 LM (*n* = 3), and matched MyoF (*n* = 6). Identical tissues were also used for the RNA-Seq analysis described above. The consensus peaks present in all replicate samples were considered for further analysis; the peak number is listed in Supplemental Table 8. Unsupervised PCA showed that MyoF samples were separated from LM samples and that G44D mut-MED12 LM (versus WT-MED12) samples distinctly clustered together (Figure 2A), consistent with the transcriptomics data (Figure 1A), suggesting that the G44D mut-MED12 may alter the chromatin interaction landscape of MED12.

To further compare the chromatin occupancy profile of MED12 between mut- and WT-MED12 LM, we used HOMER (homer.ucsd.edu/homer/ngs/mergePeaks.html) mergePeaks to identify MED12-binding sites present only in LM but not in adjacent MyoF tissues. Analysis of the genomic distribution of peaks present only in LM revealed that the percentage of MED12-binding sites at proximal promoter regions (≤1 kb) in WT-MED12 LM was 41%, which was similar to the distribution of peaks in MyoF adjacent to WT- and mut-MED12 LM (Figure 2B). Interestingly, in mut-MED12 LM, MED12-binding sites at proximal promoter regions were decreased to 15%, whereas the intronic/intergenic regions gained sites (Figure 2B).

Comparing the MED12-binding sites in intronic/intergenic regions between WT- and mut-MED12 LM revealed 3,813 sites in mut-MED12 LM only and 3,553 in WT-MED12 LM only (Figure 2C). The 3,813 mut-MED12 unique sites in intronic/intergenic regions were enriched in motifs for transcription factors of CDX4 and HOX genes (Figure 2D and Supplemental Table 9). Studies have shown that CDX4 not only regulates HOX gene expression but also modulates the WNT/β-catenin signaling pathway (59). HOX genes play important roles in development and cell differentiation and are dysregulated in several tumors (60). Recent studies have suggested a progrowth role of HOXA13 in LM (34, 37). The WNT/β-catenin signaling pathway is dysregulated in mut-MED12 LM and has a well-established role in LM growth (39, 61). The motifs enriched in 3,553 unique intronic/intergenic sites in WT-MED12 were different from those in mut-MED12 (Supplemental Table 10), and the top 5 motifs were FRA2, FRA1, JUNB, ATF3, and FOSL2 (Figure 2D). Gene Ontology (GO) analysis revealed common and distinct biological processes regulated by genes associated with unique intronic/intergenic MED12 peaks in mut- versus WT-MED12 LM (Supplemental Tables 11 and 12). Regulation of ECM organization and stem cell proliferation was overrepresented in mut-MED12 LM, whereas regulation of integrin-mediated signaling pathway and PDGF receptor pathway was overrepresented in WT-MED12 LM. Comparing the MED12 binding sites in promoter regions between WT- and mut-MED12 LM identified 305 peaks in mut-MED12 LM only and 2,179 peaks in WT-MED12 LM only (Supplemental Figure 2A), confirming that mut-MED12 LM lost MED12-binding sites in promoter regions. Although we identified unique promoter peaks in mut- and WT-MED12 LM, the top 5 motifs enriched in these peaks were similar, with NFY, SP5, and SP1 shared between mut- and WT-MED12 LM (Supplemental Figure 2B and Supplemental Tables 13 and 14). These findings suggest that mut-MED12 potentially interacts with a distinct group of transcription factors in distal enhancer regions to regulate the expression of a subset of genes involved in LM growth.

To identify potential target genes of mut-MED12, we integrated RNA-Seq data of genes only differentially expressed in mut-MED12 LM versus adjacent MyoF and ChIP-Seq data of MED12-binding sites in mut-MED12 LM. The MED12-binding sites in mut-MED12 LM were annotated to 8,834 unique genes, and 913 of these genes were significantly differentially expressed between mut-MED12 LM versus matched MyoF (Figure 2E). The top 10 up- and downregulated genes (based on fold change) were shown in Figure 2F (Supplemental Table 15) and Supplemental Figure 2C (Supplemental Table 16), respectively. TDO2 was the highest upregulated gene in mut-MED12 LM compared with MyoF (Figure 2F). Functionally, response to xenobiotic stimulus was the second highest enriched GO biological processes in these 913 genes (Figure 2G and Supplemental Table 17). Furthermore, we also performed KEGG pathway analysis using DAVID and found that the Trp metabolism pathway was one of the pathways significantly (*P* < 0.05) enriched in these 913 genes, in addition to the pathways known to be involved in LM growth such as PI3K/AKT pathway, WNT signaling, and wound healing (39, 61) (Supplemental Table 18). TDO2 catalyzes Trp breakdown to Kyn, which functions as an endogenous ligand to activate AHR (42–46), the transcription factor mediating the toxic and carcinogenic effects of a wide variety of environmental contaminants (47–49). Therefore, these findings suggest that TDO2 expression is upregulated and that its metabolites may activate the AHR signaling in mut-MED12 LM.

### mut-MED12 stimulates TDO2 expression and Kyn production in primary LM cells.

**Figure 3:**
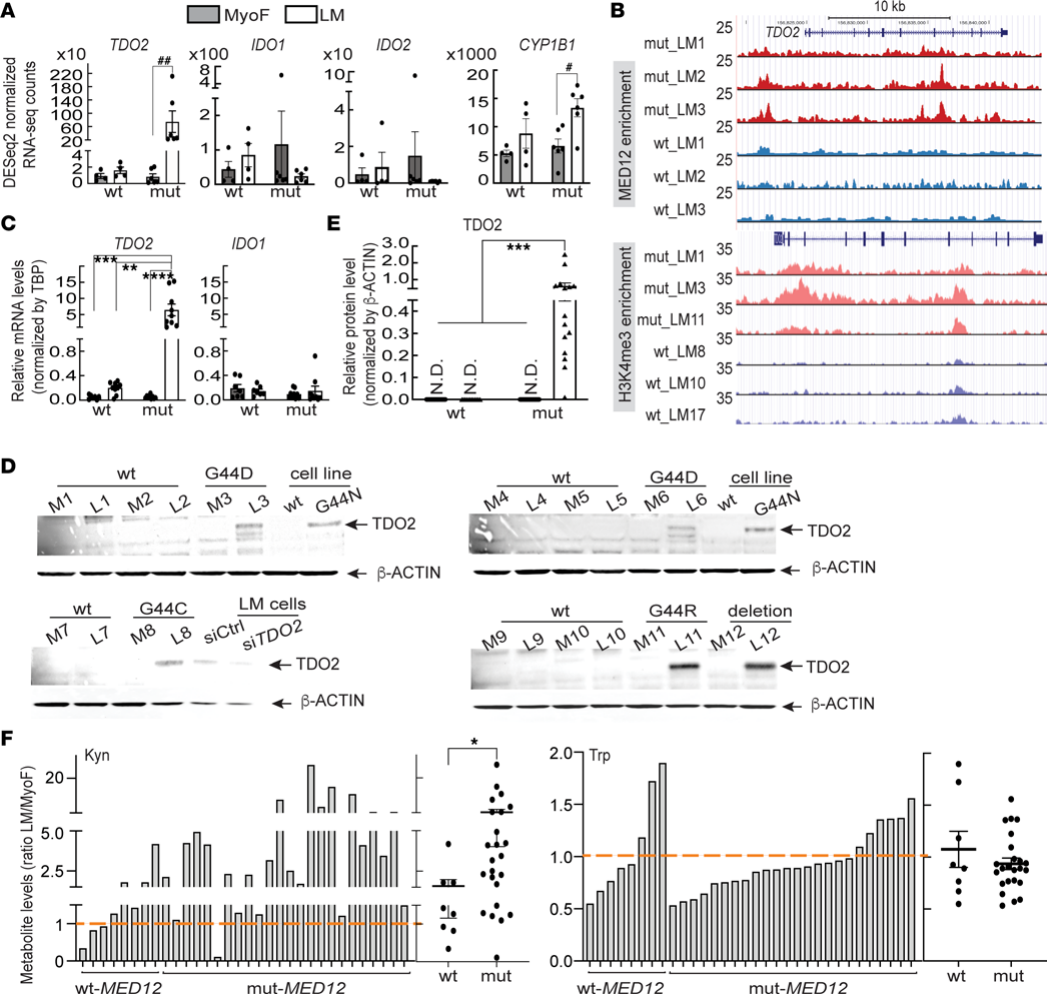
mut-MED12 stimulates TDO2 expression and Kyn production in primary LM cells. (**A**) Bar graph showing normalized RNA-Seq counts of TDO2, IDO1, IDO2, and CYP1B1 in WT-MED12 LM (*n* = 4), mut-MED12 LM (*n* = 6), and matched MyoF (*n* = 10) tissues. The default settings of DESeq2 package were used to normalize the counts. (**B**) Genome browser track view of ChIP-Seq data showing enrichment of MED12 and H3K4me3 in the TDO2 gene locus in mut- versus WT-MED12 LM (*n* = 3 of each genotype). (**C**) qPCR quantification of mRNA levels of TDO2 and IDO1 in WT-MED12 LM (*n* = 7), mut-MED12 LM (*n* = 9), and matched MyoF (*n* = 16). (**D** and **E**) Representative western blot images (**D**) and ImageJ quantification (**E**) showing higher TDO2 protein levels in mut-MED12 LM (*n* = 15) than in WT-MED12 LM (*n* = 13) and matched MyoF (*n* = 28). Some of the matched tissues for which we had both RNA and protein available were used for qPCR analysis in **C**. The protein isolated from primary mut-MED12 LM cells transfected with control siRNA or TDO2 siRNA, and from G44N mut-MED12 and the control WT-MED12 cell lines (see ref. 83 for detailed information of the cell lines), was used to show TDO2 band specificity. The images of other samples are shown in Supplemental Figure 3C; see complete unedited blots in the supplemental material. N.D., not detected; L, LM; M, MyoF. (**F**) Kyn and Trp levels in WT-MED12 LM (*n* = 8), mut-MED12 LM (*n* = 24), and matched MyoF (n=32) tissues were quantified by HPLC-MS/MS. Data are normalized to the metabolite levels in MyoF (red dotted line). Scattered dot plots adjacent to the bar graphs show the mean levels in LM in each genotype. *P < 0.05, **P < 0.005, ***P < 0.001, ****P < 0.0005, ^#^*P*_adj_ = 1.83 × 10^–10^, ^##^*P*_adj_ = 1.4 × 10^–62^ by DESeq2 (**A**), 2-way ANOVA with Tukey’s multiple-comparison test (**C** and **E**), or unpaired *t* test (**F**).

Given the progrowth role of the TDO2/Kyn/AHR pathway in various tumors (42, 50–55), we focused on investigating the role of this pathway in regulating LM cell function. As expected, the normalized RNA-Seq counts for TDO2 were dramatically higher in mut-MED12 LM compared with WT-MED12 LM and matched MyoF tissues (94-fold, *P*_adj_ = 1.4 × 10^–62^; Figure 3A). On the other hand, RNA-Seq–based gene expression levels of IDO1 and IDO2, two other Trp metabolizing enzymes, were not different between MyoF and LM (Figure 3A). Analysis of 3 previously published RNA-Seq data sets also confirmed the differential expression of TDO2, but not IDO1 or IDO2, between WT-MED12 LM, mut-MED12 LM, and their matched MyoF tissues (Supplemental Figure 3A) (34, 36, 37). In addition to G44D mut-MED12 LM, these 3 RNA-Seq data sets also included LM expressing other MED12 mutation subtypes, including G44S, G44V, G44R, L36R, G44C, deletion, and IVS 1-8 T>A (16). ChIP-Seq data demonstrate that MED12 occupancy was enriched in the TDO2 gene promoter and body regions in mut-MED12 LM (Figure 3B). We analyzed the published ChIP-Seq data for H3K4me3 modification (the mark commonly found in the promoters of actively transcribed genes) (37) and found its enrichment around TDO2 promoter region in mut- but not WT-MED12 LM (Figure 3B), supporting the enhanced TDO2 expression in mut-MED12 LM.

As shown in Figure 3C and Supplemental Figure 3B, qPCR assay of additional samples confirmed that TDO2 gene expression was significantly higher in mut-MED12 LM compared with MyoF (123-fold, 6.45 ± 1.82 versus 0.05 ± 0.01, *P* < 0.0005) and WT-MED12 LM (26-fold, 6.45 ± 1.82 versus 0.25 ± 0.02, *P* < 0.005), whereas IDO1 expression was low and not differentially expressed between LM and MyoF, and IDO2 expression was undetectable in 28 of 32 tissues. Next, we performed Western blot to determine the TDO2 protein levels in mut- and WT-MED12 LM and MyoF tissues and confirmed the upregulated TDO2 protein levels not only in LM of G44D mut-MED12 but also in those of other MED12 mutation subtypes, including G44R, Q43P, deletion, G44C, and G44A (*P* < 0.001; Figure 3, D and E, and Supplemental Figure 3C). Interestingly, mut-MED12 LM tissue also showed significantly higher mRNA levels of CYP1B1 (*P*_adj_ = 1.83 × 10^–10^; Figure 3A and Supplemental Figure 3A), the classical marker of AHR activation and the enzyme that catalyzes the conversion of estradiol to depurinating and carcinogenic metabolites (55, 62, 63). Consistent with higher TDO2 expression in mut-MED12 LM, the levels of Kyn, the catalytic product of Trp by TDO2, were higher in 23 of 24 mut-MED12 LM samples compared with MyoF (Figure 3F). The mean Kyn level in mut-MED12 LM was significantly higher than that in WT-MED12 LM (*P* < 0.05; Figure 3F). Although Trp levels were lower in 18 of 24 mut-MED12 LM versus MyoF, the difference of mean Trp level between mut- and WT-MED12 LM was not statistically significant (Figure 3F). In contrast, 5 of 8 WT-MED12 LM showed higher Kyn levels; 3 of these 5 LM samples also had higher levels of Trp compared with matched MyoF. MED12 mutations of various subtypes were included in this metabolomics study, including G44D, G44S, G44V, G44R, G44C, insertion, and IVS 1-8 T>A. Together, these data suggest that mut-MED12 may stimulate TDO2 expression, Trp metabolism, and Kyn production in mut-MED12 LM cells.

**Figure 4:**
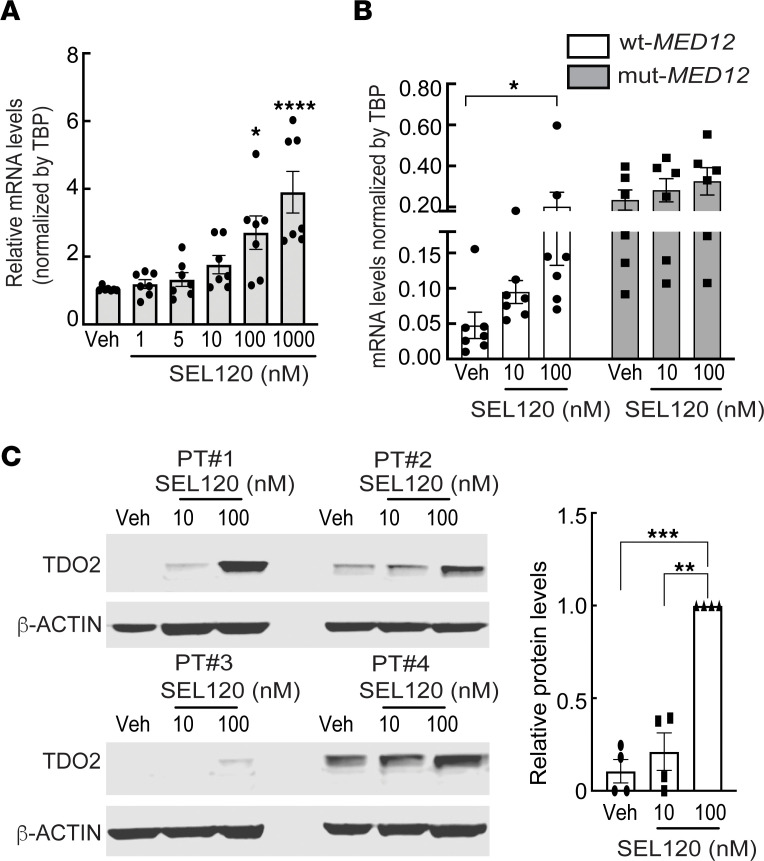
CDK8 inhibitor stimulates TDO2 expression in primary MyoF and LM cells. (**A**) Effects of different doses of CDK8 inhibitor (SEL120-34A) treatment for 24 hours on TDO2 mRNA levels in MyoF cells (*n* = 7). (**B**) Effects of CDK8 inhibitor treatment for 24 hours on TDO2 mRNA levels in mut- and WT-MED12 LM cells (*n* = 6-7). (**C**) Immunoblot images and ImageJ quantification of TDO2 protein levels in WT-MED12 LM cells treated with CDK8 inhibitor for 24 hours (*n* = 4). See complete unedited blots in the supplemental material. *P < 0.05, **P < 0.01, ***P < 0.005, ****P < 0.0001 by 1-way ANOVA with Tukey’s multiple-comparison test.

We further investigated the potential mechanism underlying mut-MED12-induced TDO2 expression. MED12 associates with a kinase module of the mediator complex that contains CDK8, which inhibits or stimulates gene transcription by modulating the general transcription factors TFIIH and RNA polymerase II (64–68). Multiple studies have reported that WT-MED12 is essential for CDK8 activity and that LM-linked MED12 mutations disrupt its activity (29–33). We determined whether direct inhibition of CDK8 kinase activity affected TDO2 gene expression. Since we observed upregulated TDO2 expression and Kyn production in LM expressing different MED12 mutations, we included all MED12 mutation subtypes in the following studies. Primary MyoF cells, which have been shown to only express WT-MED12 (16), were treated with different doses (1, 5, 10, 100, and 1,000 nM) of a CDK8-specific inhibitor (SEL120-34A) for 24 hours and TDO2 mRNA levels were quantified (69). The CDK8 inhibitor dose-dependently increased TDO2 expression (Figure 4A and Supplemental Figure 4A). The CDK8 inhibitor also significantly increased TDO2 expression in WT-MED12 but not mut-MED12 LM cells (*P* < 0.05; Figure 4B and Supplemental Figure 4B). The induction of TDO2 gene expression by the CDK8 inhibitor was confirmed at a protein level by Western blot (Figure 4C). These findings suggest that mut-MED12 stimulates TDO2 expression, at least in part, through disruption of CDK8 activity.

### TDO2-mediated Trp metabolism activates the AHR pathway in LM cells.

**Figure 5:**
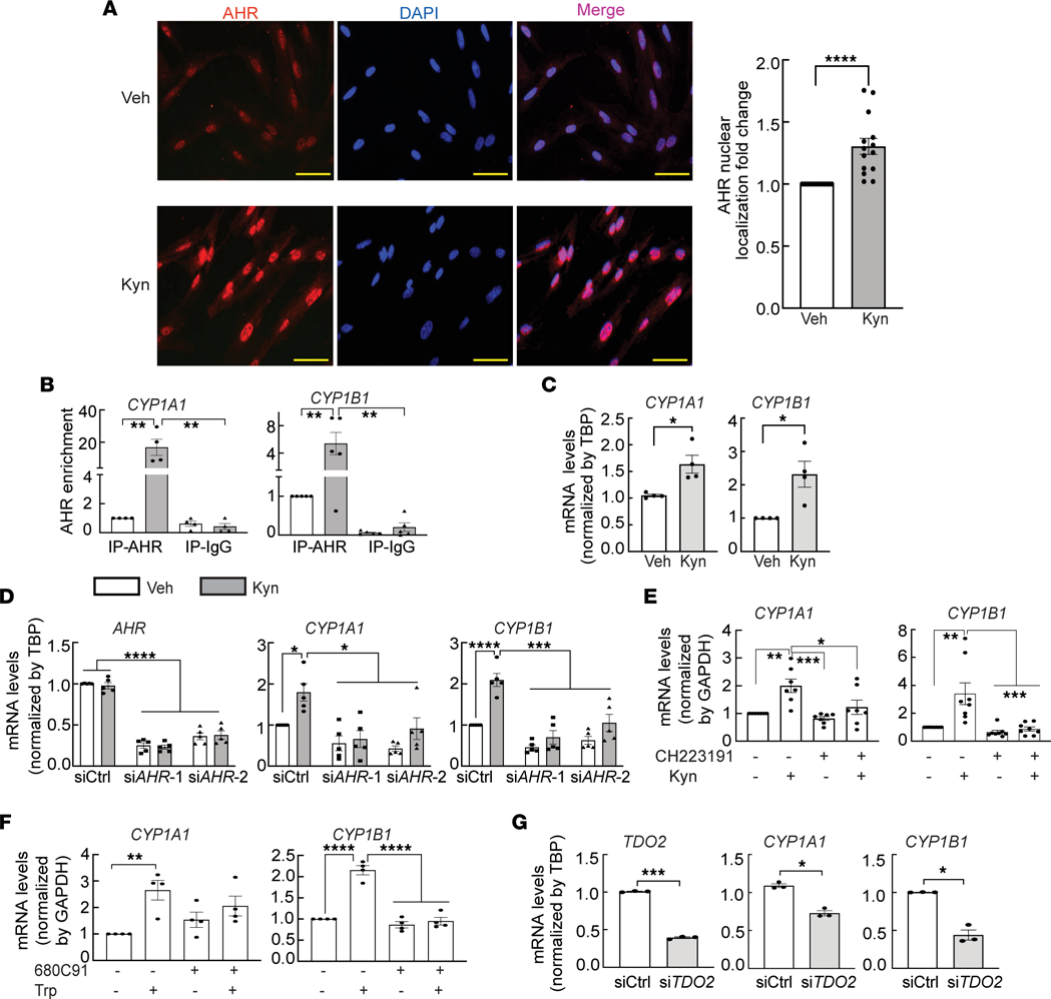
TDO2-mediated Trp metabolism activates the AHR pathway in LM cells. (**A**) AHR nuclear localization (red) was assessed by immunostaining LM cells treated with 200 μM Kyn for 24 hours. Scale bars: 200 μm. DAPI was used for nuclear staining. ImageJ was used to quantify the nuclear mean fluorescence intensity of AHR. (**B**) ChIP-qPCR assay of AHR binding to the enhancer regions of CYP1A1 and CYP1B1 genes in LM cells treated with Kyn (200 μM) for 90 minutes (*n* = 5). (**C**) qPCR of mRNA levels of CYP1A1 and CYP1B1 in LM cells treated with Kyn (200 μM) for 24 hours (*n* = 4). (**D** and **E**) Effects of AHR siRNA (**D**; *n* = 5) or AHR antagonist (**E**; CH223191, 10 μM, *n* = 7–8) on Kyn-induced CYP1A1 and CYP1B1 gene expression. Ctrl, control; si, siRNA. (**F** and **G**) Effects of TDO2 inhibitor (**F**; 680C91, 10 μM, *n* = 4) or TDO2 siRNA (**G**; *n* = 3) on Trp-induced CYP1A1 and CYP1B1 expression. *P < 0.05, **P < 0.01, ***P < 0.005, ****P < 0.0001 by paired *t* test (**A**, **C**, and **G**), 1-way (**E** and **F**), or 2-way (**B** and **D**) ANOVA with Tukey’s multiple-comparison test.

Next, we tested whether Kyn can activate AHR in LM cells. Since we did not notice significant differences between mut- and WT-MED12 LM cells with respect to their response to Kyn treatment, the data about the effects of Kyn on AHR nuclear localization, and its target gene expression, cell viability, and apoptosis in these 2 genotypes, were combined for the analysis of the following studies. Immunocytochemical staining demonstrated that treatment of primary LM cells with Kyn increased AHR nuclear localization compared with vehicle-treated cells (Figure 5A). ImageJ (NIH) quantification confirmed that the nuclear mean fluorescence intensity of AHR was significantly increased in Kyn- versus vehicle-treated cells (*P* < 0.0001; Figure 5A). We performed ChIP-qPCR to evaluate whether Kyn treatment of LM cells affected the binding of AHR to the CYP1A1 and CYP1B1 gene enhancer regions (2 known AHR target genes), which contain a canonical xenobiotic response element (XRE) (55, 62). Indeed, Kyn treatment increased AHR binding to CYP1A1 (16-fold, *P* < 0.01) and CYP1B1 (4-fold, *P* < 0.01) enhancer regions compared with vehicle-treated cells (Figure 5B). As expected, Kyn treatment significantly induced the expression of CYP1A1 (*P* < 0.05) and CYP1B1 (*P* < 0.05) as quantified by qPCR (Figure 5C and Supplemental Figure 5A). Next, we knocked down endogenous AHR using siRNA to determine whether Kyn regulates CYP1A1 and CYP1B1 gene expression through AHR. Two different AHR siRNAs (siAHR-1 and siAHR-2) significantly reduced AHR expression levels (*P* < 0.0001; Figure 5D and Supplemental Figure 5B). Kyn significantly upregulated CYP1A1 (*P* < 0.05) and CYP1B1 (*P* < 0.0001) mRNA levels, and the effect was abolished by AHR knockdown (Figure 5D and Supplemental Figure 5B). Treatment of cells with the AHR-specific antagonist CH223191 also inhibited Kyn-mediated CYP1A1 (*P* < 0.005) and CYP1B1 (*P* < 0.005) gene expression (Figure 5E and Supplemental Figure 5C). The findings indicate that Kyn activates the AHR pathway in LM cells.

Because Kyn is the downstream metabolite of TDO2-mediated Trp breakdown, we then tested whether Trp regulates AHR activity in LM cells and whether it is affected by siRNA knockdown or pharmacological inhibition of TDO2. We used primary cells from mut-MED12 LM to test the potential role of TDO2 in regulating AHR activity, owning to the upregulated TDO2 expression in mut-MED12 LM. Treatment of primary LM cells with 70 μM Trp for 48 hours in Trp-free medium significantly upregulated the mRNA levels of CYP1A1 (*P* < 0.01) and CYP1B1 (*P* < 0.0001), which were abolished by cotreatment with the TDO2 specific inhibitor 680C91 (Figure 5F and Supplemental Figure 5D). As expected, TDO2 knockdown also markedly downregulated CYP1A1 (*P* < 0.05) and CYP1B1 (*P* < 0.05) gene expression (in culture medium containing 44 μM Trp) (Figure 5G and Supplemental Figure 5E). Together, these findings suggest that TDO2-mediated Trp metabolism and Kyn production activate the AHR pathway in LM cells.

### TDO2-mediated Trp metabolism stimulates cell growth more significantly in mut-MED12 versus WT-MED12 LM.

**Figure 6:**
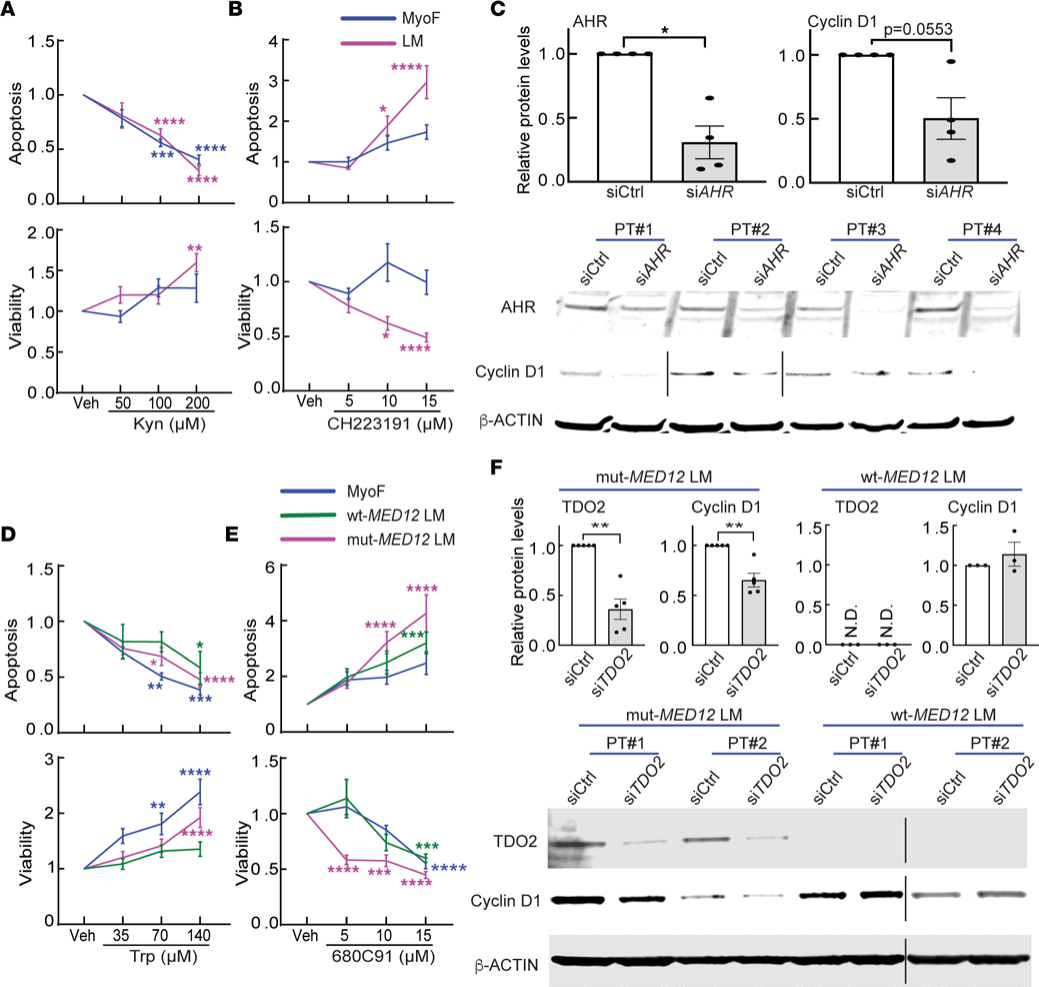
TDO2-mediated Trp metabolism stimulates cell growth more significantly in mut-*MED12* versus WT-*MED12* LM cells. (**A** and **B**) MyoF or LM cells were treated with Kyn (**A**) or the AHR-specific antagonist CH223191 (**B**) at different doses for 48 hours and analyzed with Caspase-Glo 3/7 and CCK-8 assays for cell apoptosis and viability, respectively (*n* = 5 for each tissue type). (**C**) LM cells were transfected with AHR siRNA for 72 hours, and the protein levels of AHR and Cyclin D1 were assessed by immunoblot (*n* = 4). Since Cyclin D1 protein levels variate between different patient samples, the Cyclin D1 blots were exposed at different lengths (indicated by black line) for different subjects to make the difference between AHR siRNA and control siRNA more apparent. See complete unedited blots in the supplemental material. (**D** and **E**) MyoF, WT-MED12 LM, and mut-MED12 LM cells were treated with Trp (**D**) or the selective TDO2 inhibitor 680C91 (**E**) at different doses for 48 hours and analyzed for apoptosis and viability (*n* = 5 for each tissue type). (**F**) WT- and mut-MED12 LM cells were transfected with TDO2 siRNA for 72 hours, and the protein levels of TDO2 and Cyclin D1 were assessed by immunoblot (*n* = 3–5). Samples for WT-MED12 LM of patient no. 2 (PT#2) were run on a different gel and sliced together (indicated by black line). The full unedited gel images are shown in Supplemental Data. N.D., not detected. *P < 0.05, **P < 0.01, ***P < 0.005, ****P < 0.0001 by 2-way ANOVA with Tukey’s multiple-comparison test (**A**, **B**, **D**, and **E**) or paired *t* test (**C** and **F**).

Kyn has been shown to promote growth in an AHR-dependent manner in several types of cancer cells, including colon and breast cancer cells (42, 50, 51, 70, 71); therefore, we assessed the functional importance of Kyn and the AHR activation in primary LM cells. Treatment with Kyn (50, 100, 200 μM) for 48 hours dose-dependently decreased apoptotic cell death in both primary LM and primary MyoF cells as measured by cleaved caspase activity (*P* < 0.0001; Figure 6A). The same treatment significantly increased the number of viable LM but not MyoF cells at the highest dose by Cell Counting Kit-8 (CCK-8) assay (*P* < 0.01; Figure 6A). Treatment with the AHR-selective antagonist CH223191 (5, 10, 15 μM) significantly increased apoptosis (*P* < 0.0001) and reduced viability (*P* < 0.0001) in LM cells without significant effect on MyoF cells (Figure 6B). Furthermore, knockdown of AHR decreased Cyclin D1 protein levels in LM cells (*P* = 0.0553; Figure 6C). These data suggest that Kyn promotes LM cell growth, at least in part, via activation of AHR.

Because TDO2 expression was strikingly higher in mut-MED12 LM compared with WT-MED12 LM or adjacent MyoF, we next evaluated whether mut- or WT-MED12 LM cells respond differentially to Trp or TDO2 inhibition with respect to cell viability and apoptosis. Trp treatment significantly inhibited apoptosis (70 and 140 μM; *P* < 0.0001) and increased the number of viable cells (140 μM; *P* < 0.0001) in mut-MED12 LM cells; however, only the highest dose mildly decreased apoptosis (140 μM; *P* < 0.05) in WT-MED12 LM cells (Figure 6D). In the presence of sufficient amount of Trp (180 μM), the TDO2 specific inhibitor 680C91 dramatically increased apoptosis at 10 and 15 μM (*P* < 0.0001) and decreased cell viability at all doses (5, 10, 15 μM; *P* < 0.0001) in mut-MED12 LM cells (Figure 6E). In contrast, in WT-MED12 LM cells, only the highest TDO2 inhibitor dose (15 μM) elicited these effects (Figure 6E). Although Trp significantly promoted cell viability (*P* < 0.0001) and reduced apoptosis (*P* < 0.005) in MyoF (Figure 6D), TDO2 inhibitor treatment did not significantly affect MyoF cell apoptosis or viability (except at the highest dose 15 μM) (Figure 6E). The same phenotypes were observed when the cells were treated with a different TDO2-specific inhibitor, LM10 (Supplemental Figure 6). In addition, TDO2 knockdown significantly decreased Cyclin D1 protein levels in mut-MED12 (*P* < 0.01) but not WT-MED12 LM cells (Figure 6F). Together, these data suggest that mut-MED12 LM cells likely rely on TDO2-mediated oncometabolites for survival and growth.

### Progesterone receptor regulates key genes in AHR pathway in primary LM cells.

**Figure 7:**
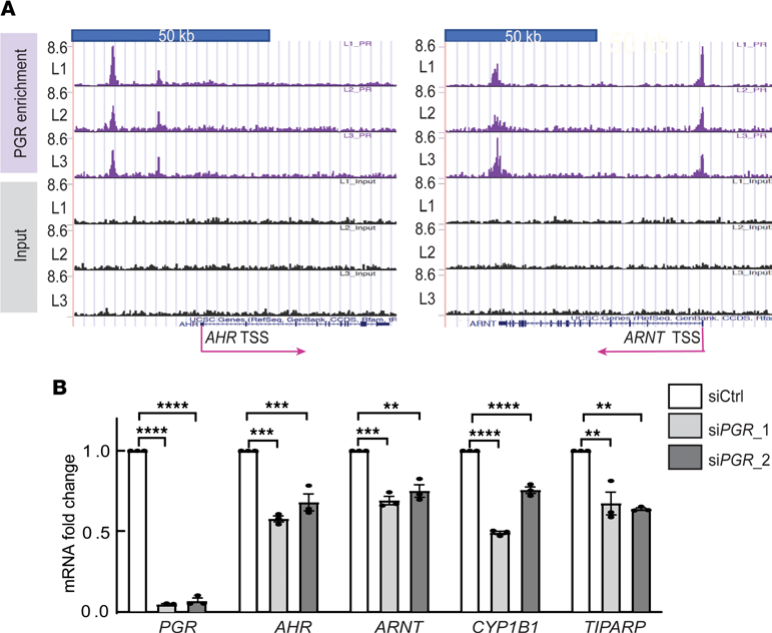
Progesterone receptor regulates key genes in the AHR pathway in primary LM cells. (**A**) Genome browser track view of ChIP-Seq data showing enrichment of PGR binding in promoter and distal enhancer regions of the AHR and ARNT genes. (**B**) Knockdown of PGR decreased mRNA levels of AHR, ARNT, and the AHR target genes CYP1B1 and TIPARP in LM cells (*n* = 3). **P < 0.01, ***P < 0.0005, ****P < 0.0001 by 1-way ANOVA with Tukey’s multiple-comparison test.

The ovarian hormone progesterone (P4), through its receptor PGR, is essential for LM growth (72); thus, we examined a possible link between PGR and the AHR pathway components in LM cells. Ligand-activated AHR translocates to the nucleus, where it forms a complex with AHR nuclear translocator (ARNT); this complex binds DNA to regulate gene expression (47–49). Analyzing our published ChIP-Seq data in LM tissues demonstrated that PGR was enriched in the promoter regions of the ARNT and AHR genes (Figure 7A) (73). Analyzing RNA-Seq data in primary LM cells after PGR knockdown in the presence of R5020 (1 × 10^–7^ M) demonstrated that PGR depletion decreased mRNA levels of ARNT (*P* < 0.0005) and AHR (*P* < 0.0005) and their downstream target genes CYP1B1 (*P* < 0.0001) and TIPARP (*P* < 0.01) (Figure 7B) (73), suggesting that PGR may affect the AHR pathway activity in LM cells by stimulating ARNT and AHR expression.

### TDO2 mRNA level positively correlates with the expression of genes critical for LM growth in vivo.

**Figure 8:**
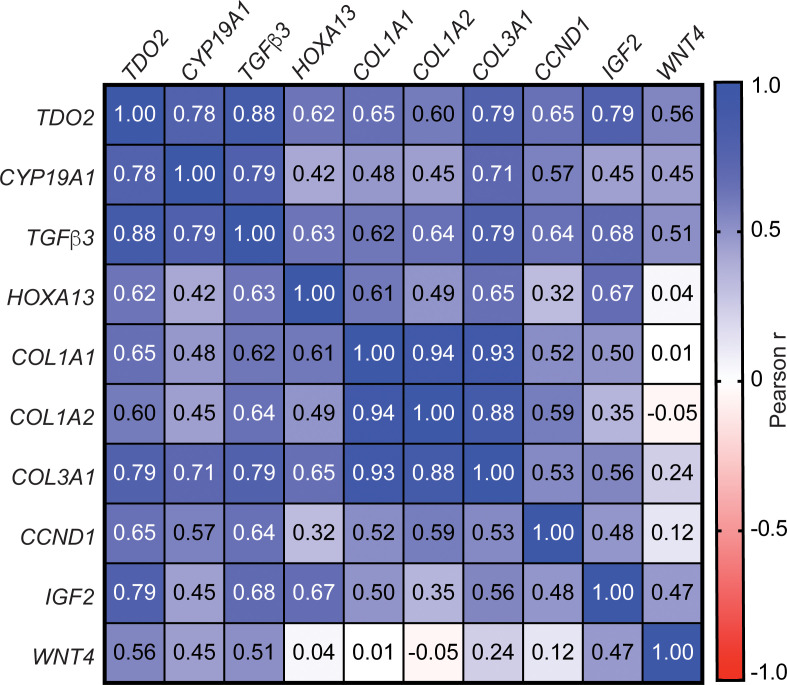
*TDO2* mRNA level positively correlates with the expression of genes critical for LM growth in vivo. Pearson’s correlation between TDO2 mRNA level and the mRNA levels of CYP19A1, TGFb3, HOXA13, COL1A1, COL1A2, COL3A1, CCND1, IGF2, and WNT4. The number in each cell represents Pearson’s *R* obtained from analyzing the log_2_-transformed RNA-Seq counts (normalized counts +1) in LM and matched MyoF tissues (*n* = 20).

To determine the potential functional significance of TDO2 overexpression in LM growth in human tissue samples, we analyzed the correlation of TDO2 mRNA level with the mRNA levels of a subset of genes crucial for cell proliferation and LM growth, including CYP19A1, TGFb3, HOXA13, COL1A1, COL1A2, COL3A1, CCND1, IGF2, and WNT4 (34, 37, 74–80) using normalized RNA-Seq counts in LM and matched MyoF tissues (*n* = 20). Pearson correlation analysis indicated a positive and highly significant correlation between TDO2 and CYP19A1 (*R* = 0.78, *P* = 5.96 × 10^–5^), TGFb3 (*R* = 0.88, *P* = 3.3 × 10^–7^), HOXA13 (*R* = 0.62, *P* = 0.003), COL1A1 (*R* = 0.65, *P* = 0.002), COL1A2 (*R* = 0.60, *P* = 0.005), COL3A1 (*R* = 0.79, *P* = 3.95 × 10^–5^), CCND1 (*R* = 0.65, *P* = 0.002), IGF2 (*R* = 0.79, *P* = 3.48 × 10^–5^), and WNT4 (*R* = 0.56, *P* = 0.011) (Figure 8). These findings further suggest that TDO2 overexpression may play an important role in LM growth in vivo.

## Discussion

We demonstrated that the most common G44D mut-MED12 affects the global MED12 binding profile in LM. Particularly, we found that mut-MED12 bound preferentially to the intronic/intergenic regions versus proximal promoter regions. TDO2 expression and its catabolic product Kyn were strikingly upregulated in mut-MED12 LM compared with WT-MED12 LM. Functionally, we demonstrated that Trp or Kyn treatment activated the AHR pathway and stimulated primary LM cell growth, and mut-MED12 LM cells showed higher sensitivity to the inhibition of TDO2 function than WT-MED12 LM cells. Additionally, PGR regulates the expression of AHR and ARNT, the key components of the AHR pathway, in LM cells. In LM tissues in vivo, TDO2 mRNA level positively correlates with the expression of progrowth genes in LM, including CYP19A1, TGFb3, HOXA13, COL1A1, COL1A2, COL3A1, CCND1, IGF2, and WNT4. Thus, the TDO2/Kyn/AHR pathway appears to be crucial for LM tumorigenesis, especially for mut-MED12 subtype tumors.

Our genome-wide analyses identified TDO2 as a target gene dramatically upregulated by mut-MED12, consistent with previous reports (40, 41). Supporting its increased transcriptional activity in mut-MED12 LM, active histone modification mark H3K4me3 was enriched at the gene promoter region of TDO2 in mut-MED12 LM (37). Although we initially used G44D mut-MED12 LM tissue samples for the genome-wide (ChIP-Seq and RNA-Seq) studies, in our follow-up work, we included LM of various MED12 mutation subtypes, including G44A, G44V, G44C, G44R, G44S, insertion, and IVS 1-8 T>A (Supplemental Table 1) (16). All the mutations change the evolutionarily conserved N-terminal amino acid sequence of MED12 that is critical for CDK8/19 activity and kinase module function (16, 33). Therefore, mut-MED12 may affect gene transcription through the disruption of CDK8 kinase activity (29–33). Supporting this notion, we found increased TDO2 expression and Kyn production not only in G44D mut-MED12 LM but also in other MED12 mutation subtype tumors such as G44S, G44V, and G44R. Furthermore, a CDK8 specific inhibitor induced TDO2 expression in primary MyoF and LM cells expressing WT-MED12 (Figure 4 and Supplemental Figure 4). Functionally, Muralimanoharan et al. recently reported that the phenotype of mut-MED12 LM–linked aberrant R-loop–induced replication stress could be recapitulated in cultured uterine smooth muscle cells following chemical inhibition of mediator-associated CDK8/19 kinase activity (81). These findings suggest that altered CDK8 kinase activity, due to site-specific mutations within MED12, has important implications in dysregulated gene expression and tumor growth in uterine LM. Considering the broad and complex effect of CDK8 kinase activity on cell function, further work needs to be done to identify the kinase substrate of the CDK8 submodule at the chromatin level in LM cells.

TDO2 catalyzes the critical step of Trp metabolism toward Kyn production. Using unbiased global metabolomics profiling, we and Heinonen et al. show that the levels of Trp, the substrate of TDO2, were decreased in mut-MED12 LM compared with MyoF, indicating its higher consumption (82). Consequently, Kyn, the catabolic product of TDO2, was dramatically increased in mut- versus WT-MED12 LM. The gene expression levels of 2 other enzymes IDO1 and IDO2 catalyzing the same step of Trp metabolism were low and not differentially expressed between LM and MyoF (41), suggesting an indispensable role of TDO2 in dysregulated Trp metabolism in mut-MED12 LM. Our group recently engineered a heterozygous MED12 G44N mutation in an immortalized uterine smooth muscle cell line using CRISPR and found that G44N mut-MED12 stimulates TDO2 expression, decreases cellular Trp, and increases Kyn levels (83). Therefore, it is plausible to speculate that replacement of G44 in MED12 activates TDO2 expression, TRP metabolism, and Kyn production. We further speculate that the disruption of CDK8/19 activity associated with a mutant MED12 may mediate the activation of the Trp/Kyn/AHR pathway via increased TDO2 expression (29–32).

AHR is a well-established ligand-activated transcription factor that regulates multiple downstream targets that promote tumor growth (84–88). Kyn was initially identified as an endogenous oncogenic ligand of AHR in glioblastoma in 2011 (42). Thereafter, Kyn was shown to be an endogenous oncometabolite that induces the expression of growth-promoting genes in an AHR-dependent manner in several other cancers such as colon and breast (50, 51, 70). Studies in various tumors have suggested that Kyn produced by tumor cells activates AHR and plays crucial roles in promoting tumor progression via: (a) silencing the antitumor immune response in a paracrine fashion and/or (b) acting directly on neoplastic cells by enhancing their survival and proliferation (42, 53–55, 70, 89). Using a 2D-culture system of isolated neoplastic primary LM cells, we found that mut-MED12 LM cells showed higher sensitivity compared with WT-MED12 LM cells with respect to the effects of Trp and TDO2 inhibition on cell viability and apoptosis. These findings strongly suggest that excessive Kyn production by TDO2 overexpression in mut-MED12 LM cells activates the AHR pathway in neoplastic LM cells, promoting cell survival and proliferation and leading to increased tumor growth.

Trp treatment activated the expression of AHR target genes CYP1A1 and CYP1B1, which could be blocked by TDO2 siRNA or a TDO2 specific inhibitor (Figure 5, F and G, and Supplemental Figure 5, D and E), indicating that Trp metabolites catalyzed by TDO2 activate the AHR pathway. Interestingly, knockdown of PGR decreased the mRNA levels of AHR and ARNT (Figure 7), suggesting that PGR may influence the Trp/Kyn/AHR pathway activation by stimulating or maintaining the expression of AHR and ARNT. Interaction between nuclear receptor–activated pathways is a key regulatory mechanism in gene transcription, leading to modifications in patterns of gene expression and cell fate (90, 91). Other studies have reported complex and tissue-specific interactions between ligand-activated AHR and steroid hormone receptor signaling (92). It is also plausible that the Kyn-activated AHR pathway may dysregulate the physiological functions of estradiol/ESR1 or progesterone/PGR in adjacent myometrial tissue, and this may contribute to increased LM growth. As a future direction, we will define whether and how Trp metabolism–mediated AHR pathway activation interferes with the steroid hormone signaling with respect to LM growth.

It is worth noting that human placenta expresses both IDO1 and IDO2, which are increased at term compared with the first trimester placenta (93, 94). Placental IDO activity is essential during pregnancy because it prevents immune activation and creates a tolerogenic environment for the fetus. Recently, studies suggest that the Kyn pathway alterations in the placenta are unlikely to affect circulating metabolite concentrations in the mother (95). Further studies will be needed to determine whether Trp metabolites produced by overexpression of TDO2 in mut-MED12 LM impact pregnancy and whether increased production of these metabolites during pregnancy affects LM growth.

In this study, we discovered that mut-MED12 alters the genome-wide MED12-binding landscape, leading to increased MED12 occupancy at intronic/intergenic regions. DNA methylation interferes with the interactions between DNA and specific transcription factors and chromatin proteins (96). George et al. recently reported that DNA methylation signatures can segregate MyoF from LM, and LM can be further grouped based on the gene mutation subtype (34). They also found that hypermethylated regions are enriched with binding sites for EZH2 and SUZ12, components of polycomb repressive complex 2 (PCR2), contributing to chromatin compaction and accessibility change (97). Alteration in metabolic activity may have a profound influence on chromatin structure in cancers (98). In fact, Kyn and AHR activation have been shown to alter histone modifications and chromatin accessibility (99–101). Whether the dysregulated Trp metabolism associated with TDO2 overexpression plays a causative role in altered epigenome and transcriptome in mut-MED12 LM warrants further study.

Furthermore, we found that the unique MED12-binding sites in intronic/intergenic regions of mut-MED12 LM were enriched in DNA binding motifs for transcription factors of CDX4 and HOX genes (Figure 2D), which have been implicated in LM growth (34, 37, 39, 61, 102). Studies to further clarify whether MED12 interacts with CDX4 or HOX genes at chromatin levels and whether the interaction is altered by mut-MED12 will be important in increasing our understanding of mut-MED12–linked tumor growth, which is beyond the scope of the current study. Interestingly, the genes associated with these intronic/intergenic sites were significantly enriched in functional pathways regulating stem cell proliferation (Supplemental Table 11). The findings support previous studies indicating that MED12 mutations or other genetic modifications may activate quiescent myometrium stem cells, which may serve as cellular clones for tumor development and growth (103, 104).

The positive correlation (Pearson’s) between TDO2 gene expression and the mRNA levels of CYP19A1, TGFb3, HOXA13, COL1A1, COL1A2, COL3A1, CCND1, IGF2, and WNT4 further corroborates the stimulatory role of TDO2 overexpression in LM growth. Multiple Trp metabolites can activate AHR, and TDO2/IDO-mediated Trp metabolites can mediate other progrowth pathways in tumor cells. For example, Bishnupuri et al. report that Kyn pathway metabolites activate PI3-AKT signaling to promote colon cancer cell survival and proliferation (105). In this study, we only focused on the effects of Kyn-mediated AHR activation on LM cell growth using an in vitro cell culture model. We will continue to investigate the roles of other metabolites or pathways associated with TDO2-mediated Trp metabolism in LM growth and validate our in vitro findings using the LM xenograft mouse model in vivo (72). Our studies suggest that dysregulation of Trp metabolism caused by TDO2 overexpression could be the key factor of mut-MED12–associated LM growth. We do not anticipate that all mut-MED12 tumors would respond to TDO2 treatment and shrink, since tumor biology is highly complex and involves multiple redundant tumorigenic pathways. We, however, anticipate that a substantial number of patients with mut-MED12 tumors may benefit from this approach.

In conclusion, our findings suggest that the TDO2/Kyn/AHR pathway is activated in LM — particularly in mut-MED12 LM. Understanding the mechanistic link between TDO2 overexpression, Trp metabolism and Kyn production, AHR activation, and LM cell function alterations will not only provide critical evidence supporting a causal link between MED12 mutation and the development of LM, but it may also open new avenues toward the development of strategies preferentially targeting the mut-MED12 LM subtype and, thus, precision medicine in the area of uterine LM.

## Methods

A comprehensive description of the methods for genotyping for MED12 mutation analysis, TDO2 and AHR siRNA knockdown, RNA isolation and qPCR, cell viability assay, cell apoptosis assay, immunofluorescence staining, ChIP-qPCR, and immunoblot analysis is included in Supplemental Methods.

### Tissue collection and cell preparation.

Human MyoF and LM tissues were procured from premenopausal women (44 ± 5 years old) undergoing myomectomy or hysterectomy. Samples from patients who received hormone treatments 6 months prior to the surgery were excluded from the study. LM and MyoF tissue fragments were manually minced into small pieces (1–2 mm^3^), followed by incubation for 5–6 hours in HBSS containing 1% antibiotic/antimycotic solution (catalog 15240062, Thermo Fisher Scientific), 8 μg/mL DNase I (catalog D5025-150KU, Sigma-Aldrich), and 1.5 mg/mL collagenase (catalog C0130-1G, Sigma-Aldrich), at 37°C on a shaker. The dissociated primary cells were maintained in Smooth Muscle Cell Growth Medium (catalog CC-3182, Lonza) supplemented with 5% FBS, insulin, hFGF-B, GA-1000, hEGF, and 1% antibiotic/antimycotic in a humidified atmosphere with 5% CO_2_ at 37°C because our previous study found that this medium may reduce the loss of LM cells expressing mut-MED12 during in vitro culture (41, 106). Primary MyoF and LM cells isolated from different specimens were randomly assigned to different experiments. Several factors limited the possibility of performing all the experiments using primary cells isolated from the specimen of the same patient: (a) primary MyoF and LM cells grow slowly in vitro; (b) all primary MyoF and LM cells in this study were used within 2 passages to avoid the possible long-term in vitro culture–induced phenotype change (106, 107); and (c) limited volume of the specimen was available for research lab use.

### RNA-Seq and bioinformatics analysis.

Total RNA was isolated from approximately 30 mg of frozen LM and matched MyoF tissues using the RNeasy Fibrous Tissue kit (catalog 74704, Qiagen) following the manufacturer’s instructions. RNA quality was assessed using the Agilent Bioanalyzer 2100. RNA-Seq libraries were prepared using the Stranded RNA-Seq kit with RiboErase (catalog KK8483, KAPA Biosystems). The library concentrations were measured by Qubit 4 Fluorometer (catalog Q33238, Thermo Fisher Scientific), and library quality was determined using the Agilent Bioanalyzer 2100. The libraries were sequenced as 75 bp single-end reads for 40 million reads per sample at Northwestern University’s NUSeq Core using the NextSeq 500 system (Illumina). The quality of DNA reads was evaluated using FastQC (Babraham Bioinformatics). Adapters were trimmed, and reads of poor quality or that aligned to rRNA sequences were filtered out. The cleaned reads were aligned to the Homo sapiens Genome (hg38) using STAR aligner (v2.6.3) with default settings (108). Read counts for each gene were calculated using htseq (109), and the default settings of DESeq2 package were used to normalize the counts (median of ratios method-internal normalization using geometric mean) (110, 111). Differential gene expression was detected using DESeq2 with the cutoff of *P*_adj_ < 0.05. Expression values were transformed using DESeq2’s regularized log transformation (rlog) before visualization using PCA. After removing genes with low read count (<100 across all samples), 21,288 genes were used for the PCA using an unsupervised approach. Pearson correlation between TDO2 and candidate genes (CYP19A1, TGFb3, HOXA13, COL1A1, COL1A2, COL3A1, CCND1, IGF2, and WNT4) was analyzed using log_2_-transformed normalized counts for each gene. Since zero counts cannot be “logged,” 1 was added to each gene count prior to log_2_ transforming the data (112, 113). Enriched GO terms and pathways were identified using the DAVID (57, 58).

### ChIP-Seq and bioinformatics analysis.

In total, 0.2–0.5 g of frozen LM and matched MyoF tissues were used for ChIP-Seq. Tissue was finely ground with mortar and pestle in liquid nitrogen, followed by fixation in 1% paraformaldehyde for 15 minutes and quenching with 1× glycine for 5 minutes at room temperature. Chromatin was isolated using the SimpleChIP Enzymatic Chromatin IP Kit (catalog 9003S, Cell Signaling Technology) following the manufacturer’s protocol. A total of 15 μg of chromatin was incubated with 6 μg of MED12 antibody to immunoprecipitate the DNA, which was then purified for library preparation. Antibody information is provided in Supplemental Table 19. The libraries were prepared using the KAPA Hyper Prep Kit (catalog KK8502, KAPA Biosystems) and KAPA Single-Indexed Adapter Kit (catalog KK8722, KAPA Biosystems). The libraries were quantified, quality checked, and sequenced in the NUSeq Core. Quality control of reads was performed using FastQC. The reads were then aligned to the hg38 genome using Bowtie2 with the in-house script, and alignment information for each read is stored in the BAM format (114). Peak calling was performed using HOMER: findPeaks (-style histone). BED files were constructed from the peaks using HOMER (pos2bed function). ChIPseeker was used to annotate the BED files of each replicate to obtain the frequency of binding sites in each annotated genomic region. The BAM and BED files from replicates were used as input for the Bioconductor package DiffBind to merge the intervals of peaks and identify the consensus overlapping regions across all replicates in mut-MED12 LM, WT-MED12 LM, and their matched MyoF samples (115). The peak consensus regions in the genome were also annotated using ChIPseeker. Motif analyses were performed using HOMER (findMotifsGenome.pl, -size 200 bp). Enriched GO terms and pathways were identified using DAVID.

### Hydrophilic metabolite profiling.

Frozen MyoF or LM tissues were finely ground with a mortar and pestle in liquid nitrogen. Methanol (80%, cooled to –80°C) was added to 50 mg of the ground tissue at 20 μL/mg tissue. The tissue-methanol mixture was vortexed and 200 μL was transferred to a tube containing 800 μL 80% methanol (cooled to –80°C), followed by incubation at –80°C for at least 4 hours. Then the mixture was vortexed rigorously for 1 minute and centrifuged at 20,000*g* for 15 minutes at 4°C. The metabolite-containing supernatant was transferred to a new tube and submitted to the Northwestern University Metabolomics Core for analysis using high-performance liquid chromatography–tandem mass spectrometry (HPLC-MS/MS) as described previously (116).

### Statistics.

GraphPad Prism 9 (GraphPad Inc.) was used for statistical analyses and generating graphs. Student’s *t* test (2-tailed) or repeated-measures one-way or 2-way ANOVA was used to compare means between groups. All experiments were performed in triplicate with samples from at least 3 patients. Patient numbers (*n*) are provided in the figure legends, data points in the bar plots indicate biological replicates from different patient samples for each experiment, and data are shown as the mean ± SEM. No sample was excluded during the analysis. A *P* value less than 0.05 was considered significant. 

### Study approval.

The use of human tissue was approved by Northwestern University IRB, and written informed consent was received prior to use of samples in the study.

### Data availability.

All high-throughput sequencing data that support the findings of this study have been deposited in the GEO repository (GSE204968 and GSE204969). All data generated or analyzed during this study are included in this article or in data repositories (34, 36, 37, 73). Values for all data points in graphs can be found in the Supporting Data Values file.

## Author contributions

AZ, AJD, HW, JSC, SL, TI, OW, OS, PS, and PY performed the experiments. YH, AJD, SL, DC, PY, and YD performed bioinformatics analyses. AZ, YH, YD, DC, TGB, SEB, and PY prepared the manuscript with input from all authors. SEB and PY conceived the experiments and supervised the project.

## Supplementary Material

Supplemental data

Supplemental table 2

Supplemental table 3

Supplemental table 4

Supplemental table 5

Supplemental table 6

Supplemental table 7

Supplemental table 8

Supplemental table 9

Supplemental table 10

Supplemental table 11

Supplemental table 12

Supplemental table 13

Supplemental table 14

Supplemental table 15

Supplemental table 16

Supplemental table 17

Supplemental table 18

Supporting data values

## Figures and Tables

**Table 1:**
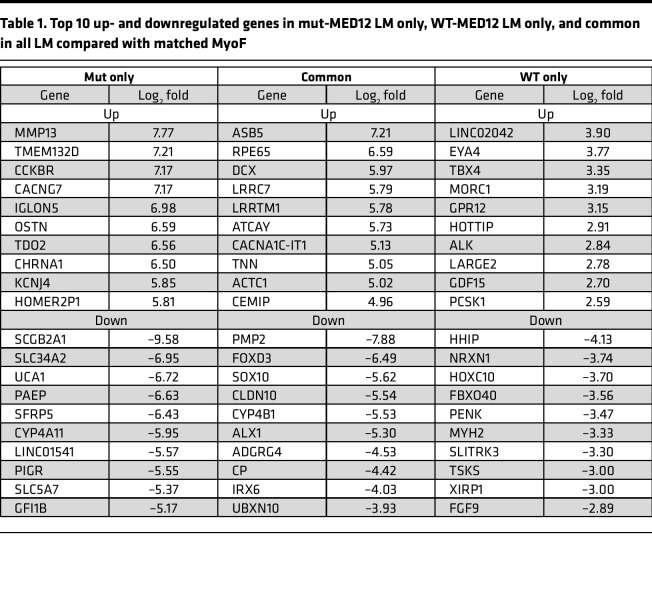
Top 10 up- and downregulated genes in mut-MED12 LM only, WT-MED12 LM only, and common in all LM compared with matched MyoF

## Version 1 08/22/2023

In-Press Preview

## Version 2 09/22/2023

Electronic publication
