# Supplementary material for: MED12 mutation activates the tryptophan/kynurenine/AHR pathway to promote growth of uterine leiomyomas
Source: JCI Insight. 2023 Sep 22;8(18):e171305. doi: 10.1172/jci.insight.171305 (PMC10561729; doi:10.1172/jci.insight.171305)

Supplemental Material for

MED12 Mutation Activates Tryptophan-Kynurenine-AHR Pathway to Promote Growth of Uterine Leiomyoma

Azna Zuberi[1*], Yongchao Huang[2*], Ariel J. Dotts[1], Helen Wei[1], John S. Coon V[1], Shimeng Liu[1], Takashi Iizuka[1], Olivia Wu[1], Olivia Sotos[1], Priyanka Saini[1], Debabrata Chakravarti[1], Thomas G Boyer[3], Yang Dai[2†], Serdar E Bulun[1†‡], Ping Yin[1†‡]

Corresponding authors:
Serdar E. Bulun: s-bulun@northwestern.edu
Ping Yin: p-yin@northwestern.edu

Supplemental Methods

Genotyping for *MED12* mutation in LM

Total RNA or genomic DNA was isolated from fresh tissues or primary cells using the AllPrep DNA/RNA mini kit (Cat no: 80204, Qiagen) per the manufacturer's instructions. Genomic DNA or cDNA was amplified using a hot start DNA polymerase kit (Cat no: 71086-3, Sigma-Aldrich) and primers as previously described (1, 2). The PCR products were purified and sequenced at AGCT Incorporated. The sequences were analyzed using Indigo software (www.gear-genomics.com/indigo) and compared with the human MED12 reference sequence from NCBI (NM_005120). Information on race/ethnicity and *MED12* mutation status for the patient samples used in this study is listed in Supplemental Table 1.

TDO2 and AHR siRNA knockdown

LM cells were transfected with 100 nM human *TDO2* siRNA, *AHR* siRNA, or non-targeting control siRNA using DharmaFECT 1 (Cat no: T-2001-02, Dharmacon) transfection reagent following the manufacturer's instructions. Information about the siRNA used is provided in Supplemental Table 19. To determine the effects of Kyn on AHR-mediated gene expression, after 24 h of transfection of *AHR* siRNA, the cells were starved with Trp- and phenol-red free Dulbecco's Modified Eagle's Medium (DMEM)/F12 medium (Cat no: CS050-01, US Biological Life Sciences) supplemented with 0.2% charcoal stripped (CS)-FBS (starvation medium) for 24 h, followed by treatment with Kyn (200 µM) or vehicle for 48 h in starvation medium. The cells were transfected with *TDO2* siRNA following the same protocol and incubated in phenol-red free DMEM/F12 medium (containing 44 µM Trp, Cat no: 11039-047, Thermo Fisher Scientific,) supplemented with 0.2% CS-FBS for 72 h before harvested for RNA isolation.

RNA isolation and real-time qPCR

Total RNA was isolated using the RNeasy mini kit (Cat no: 74106, Qiagen). cDNA was synthesized using qScript cDNA SuperMix (Cat no: 95048-100, VWR International) and mRNA levels of *TDO2, IDO1, IDO2, AHR, CYP1B1, and CYP1A1* were quantified using RT-qPCR (QuantStudio 12K Flex, Applied Biosystems Waltham) with Taqman Universal Mastermix (Cat no: 4364338, Thermo Fisher Scientific) and normalized to TATA-BOX Binding Protein (TBP), glyceraldehyde-3-phosphate dehydrogenase (GAPDH), or 18sRNA. The primers used for RT-qPCR are listed in the Supplemental Table 19. Expression levels were calculated by applying the comparative cycle threshold (Ct) method. To determine the effects of CDK8 specific inhibitor SEL120-34A (Cat no: S8840, Selleck Chemicals LLC) on TDO2 gene expression, the cells were starved in phenol-red free DMEM/F12 medium with 0.2% CS-FBS for 24 h followed by treatment with vehicle (DMSO) or different doses (1, 5, 10, 100 1000 nM) of CDK8 inhibitor for 24h before harvesting for RNA isolation and protein extraction.

Cell viability assay

The effects of Trp (Cat no: S3987, Selleck Chemicals), the TDO2 specific inhibitors 680C91 (Cat no: SML0287-5MG, Sigma-Aldrich) and LM10 (Cat no: T4410, TargetMol), Kyn (Cat no: K8625-25MG, Sigma-Aldrich), and the AHR-selective antagonist CH223191 (Cat no: T2448, TargetMol) on MyoF and LM cell viability were assessed using the Cell Counting Kit-8 (CCK-8) (Cat no: CK04-05; Dojindo) following the manufacturer's protocol. The kit utilizes a highly water-soluble tetrazolium salt, WST-8, which produces a water-soluble formazan dye upon reduction in the presence of an electron mediator. The amount of formazan generated by dehydrogenases is directly proportional to the number of living cells. Briefly, 5000-7000 primary MyoF or LM cells were seeded in 100 µl of DMEM/F12 medium containing 10% FBS and 1% antibiotic/antimycotic in a standard 96-well plate until 75-80% confluency. Then the cells were starved for 24 h and

treated with Trp (35, 70, 150 µM), 680C91 (5, 10, 15 µM), LM10 (25, 50, 100 µM), Kyn (50, 100, 200 µM), CH223191 (5, 10, 15 µM), or vehicle (Trp and Kyn were dissolved in H_2O and the others were dissolved in DMSO). To assess the effects of Trp or Kyn, the cells were treated in Trp- and phenol red-free medium containing 0.2% CS-FBS. Otherwise, the cells were treated in phenol-red free DMED/F12 medium with 0.2% CS-FBS. After 24 h of treatment, 10 µl of CCK-8 solution was added to each well and incubated for 3 h before measuring the absorbance at 450 nm using a microplate reader.

Cell apoptosis assay
Apoptosis was measured using the Caspase-Glo 3/7 Assay kit (Cat no: PRG8090, Promega) following the manufacturer's instructions. MyoF and LM cells were seeded in a white-walled 96-well plate and treated with vehicle, Kyn, CH223191, Trp, 680C91, or LM10 for 24 h, as described for the viability assay above. At the end of the treatment, 100 µl of Caspase-Glo® 3/7 reagent was added to each well followed by incubation at room temperature for 3 h. The luminescence of each sample was measured using a microplate reader.

Immunofluorescent staining
LM cells were grown on glass coverslips in 6-well plates until approximately 60% confluency. The cells were starved in Trp- and phenol red-free DMEM/F12 medium containing 0.2% CS-FBS for 24 h followed by treatment with 200 µM Kyn for 24 h. The cells were fixed with freshly prepared 4% paraformaldehyde in phosphate-buffered saline (PBS) for 10 min and permeabilized with 0.5% Triton X-100 in PBS for 5 min at room temperature. Then the cells were blocked with 5% BSA in PBS for 1 h followed by incubation with anti-AHR antibody (1:500 in 1% BSA, Supplemental Table 19) overnight at 4°C. The cells were then incubated with Alexa Fluor Plus 555 conjugated goat anti-mouse IgG (H+L) secondary antibody (Supplemental Table 19) for 1 h followed by incubation in DAPI (0.5 µg/ml, Cat no: 50-196-4625, Thermo Fisher Scientific) for 5 min at room temperature. Images were captured by a Leica DM5000 B microscope with an attached Leica DFC450 C digital microscope camera (Leica) and quantified by ImageJ.

ChIP-qPCR
LM cells were treated with 200 µM Kyn for 90 min, followed by crosslinking with 1% paraformaldehyde for 10 min and quenching with 1X glycine for 5 minutes at room temperature. Chromatin was isolated as described for ChIP-seq. Chromatin immunoprecipitation was performed using ChIP-validated AHR antibody (Supplemental Table 19). The immunoprecipitated DNA was purified and analyzed by RT-qPCR to assess the effects of Kyn treatment on AHR binding at the *CYP1A1* and *CYP1B1* gene loci using previously published primers (3). Normal rabbit IgG was used as a negative control. The data were analyzed by the percent input method.

Immunoblot analysis
LM cells were transfected with *AHR* siRNA or *TDO2* siRNA as described above for *TDO2* and *AHR* siRNA knockdown. 24 h after transfection, the cells were maintained in phenol red free DMEM/F12 with 0.2% CS-FBS for 72 h before harvested for the assay. Frozen MyoF or LM tissues were finely grounded with a mortar and pestle in liquid nitrogen. The protein from LM cells or frozen tissues was extracted using RIPA buffer containing protease inhibitor (Cat no: 11836170001, Sigma-Aldrich) and quantified using the BCA assay kit. The protein samples were prepared using 4X LDS sample buffer (Cat no: NP0007, Thermo Fisher Scientific) and electrophoresed on a 4% to 12% Novex Bis-Tris polyacrylamide precast gel (Cat no: NP0335BOX, Thermo Fisher Scientific). The protein was transferred to polyvinylidene difluoride membranes, which were incubated with primary antibodies (1:1000, AHR, TDO2, or Cyclin D1, Supplemental Table 19) at 4°C overnight. β-ACTIN was used as a loading control. The membranes were then incubated with horseradish peroxidase-conjugated secondary antibody for

1 h at room temperature. The membranes were developed using Luminata Crescendo horseradish peroxidase substrate (Cat no: WBLUR0500, Millipore Sigma) and imagined and quantified using iBright. Our group recently engineered a heterozygous *MED12* G44N mutation in an immortalized uterine smooth muscle cell line using CRISPR and found that G44N mut-MED12 stimulates TDO2 expression (4). The protein isolated from primary mut-*MED12* LM cells transfected with control siRNA or *TDO2* siRNA, and from G44N mut-*MED12* and the control wt-*MED12* cell lines was used as a control for TDO2 protein detection.

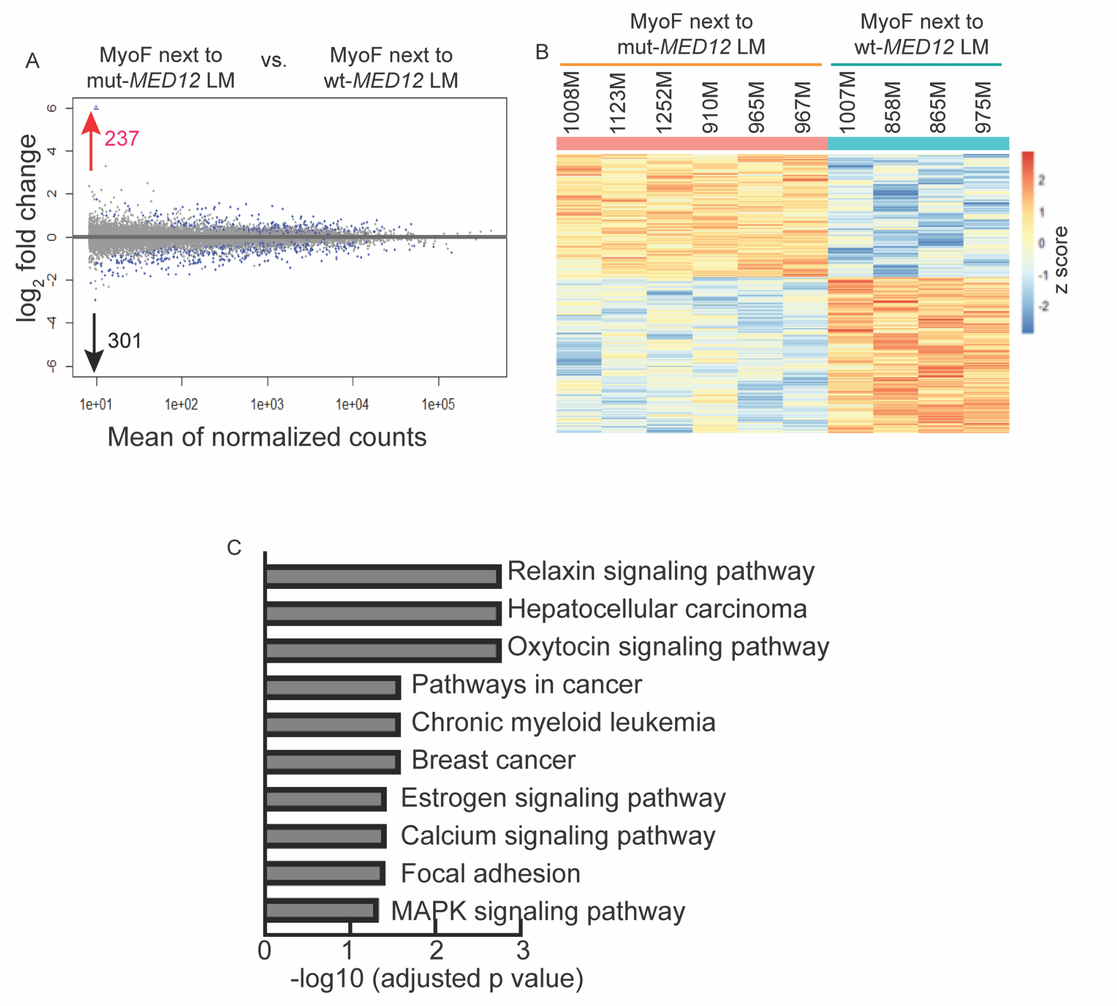

Supplemental Figure 1: (**A**) MA (log_2 fold change and means average) plot and (**B**) heat map showing changes in gene expression (up- and down-regulated) in MyoF adjacent to mut-*MED12* LM vs. MyoF adjacent to wt-*MED12* LM tissues. Gene expression levels relative to the mean expression are shown as row Z-scores. (**C**) Significantly enriched KEGG pathways in genes differentially expressed in MyoF adjacent to mut-*MED12* LM vs. MyoF adjacent to wt-*MED12* LM.

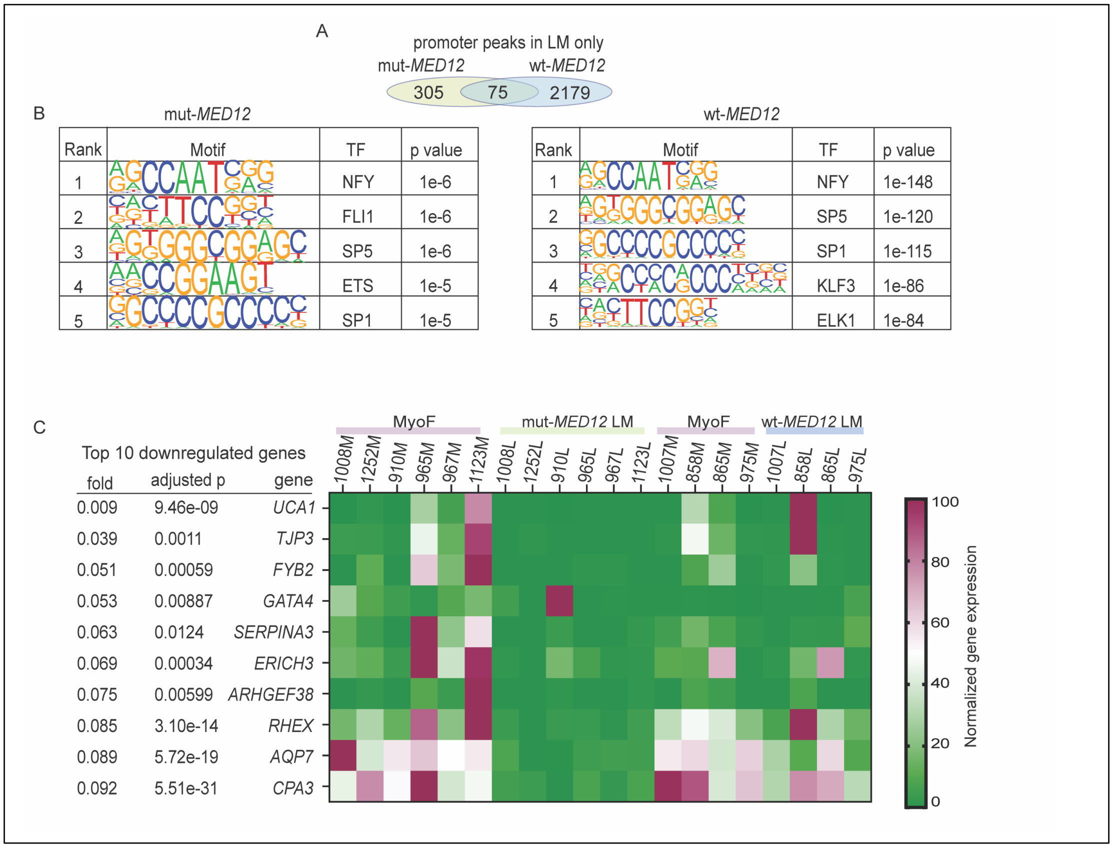

Supplemental Figure 2: (**A**) Venn diagram showing shared and unique proximal promoter MED12-binding sites between mut- and wt-*MED12* LM. (**B**) The top 5 motifs enriched in 305 promoter peaks in mut-*MED12* LM only and 2179 peaks in wt-*MED12* LM only. (**C**) Heatmap showing percentage normalized gene expression of top 10 downregulated genes among the 913 genes only differentially expressed in mut-*MED12* LM vs. MyoF and associated with adjacent MED12 binding sites shown in Figure 2E.

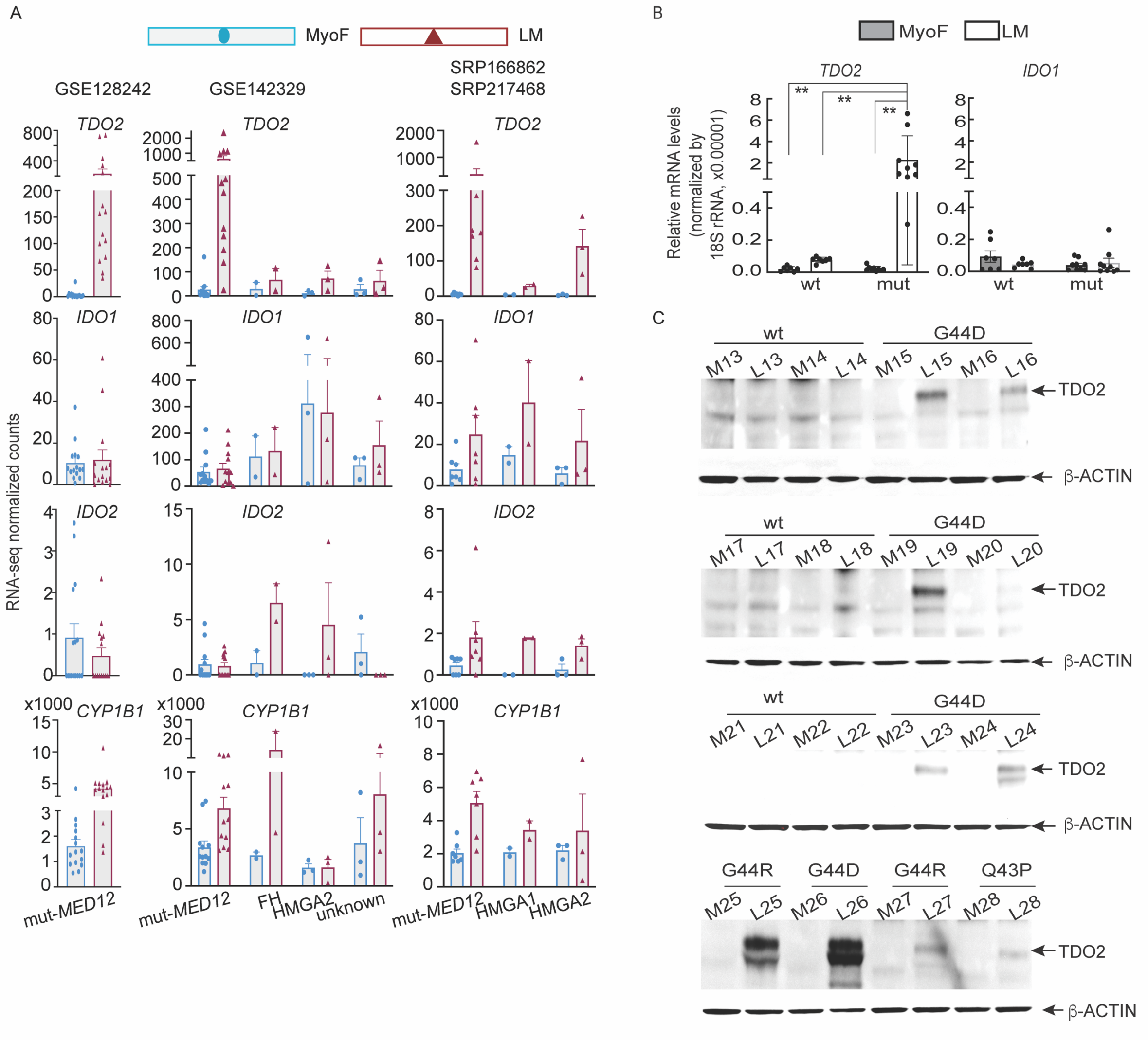

Supplemental Figure 3: (**A**) Bar graphs show normalized RNA-seq counts of genes of *TDO2*, *IDO1*, *IDO2*, and *CYP1B1* in mut-*MED12* LM and LM expressing mutations of the fumarate hydratase (*FH*) deficiency or *HMGA1* or *HMGA2* overexpression. The data were retrieved from previously published RNA-seq datasets (GSE128242, GSE142329, SRP166862, SRP217468). (**B**) RT-qPCR quantification of mRNA levels of *TDO2* and *IDO1* in wt-*MED12* LM (n=7), mut-*MED12* LM (n=9), and matched MyoF (n=16) tissues. The *TDO2* and *IDO1* mRNA levels were normalized by *18S rRNA*. **p<0.01 by two-way ANOVA with multiple comparison test. (**C**) Western blot images of additional samples for Figure 3 D and E, and the full unedited gel images were shown in Supplemental Data.

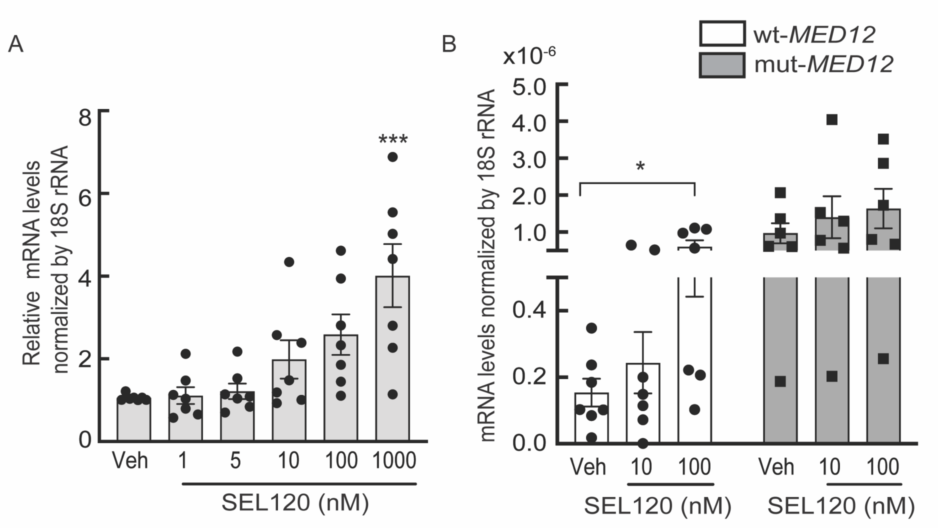

Supplemental Figure 4: CDK8 inhibitor stimulates TDO2 expression in primary MyoF and LM cells. (**A**) Effects of different doses of CDK8 inhibitor (SEL120-34A) treatment for 24 h on *TDO2* mRNA levels in MyoF cells (n=7). (**B**) Effects of CDK8 inhibitor treatment for 24 h on *TDO2* mRNA levels in mut- and wt-*MED12* LM cells (n=6-7). The *TDO2* mRNA levels were normalized by *18S rRNA*. *p<0.05 and ***p<0.005 by One-way ANOVA with multiple comparison test.

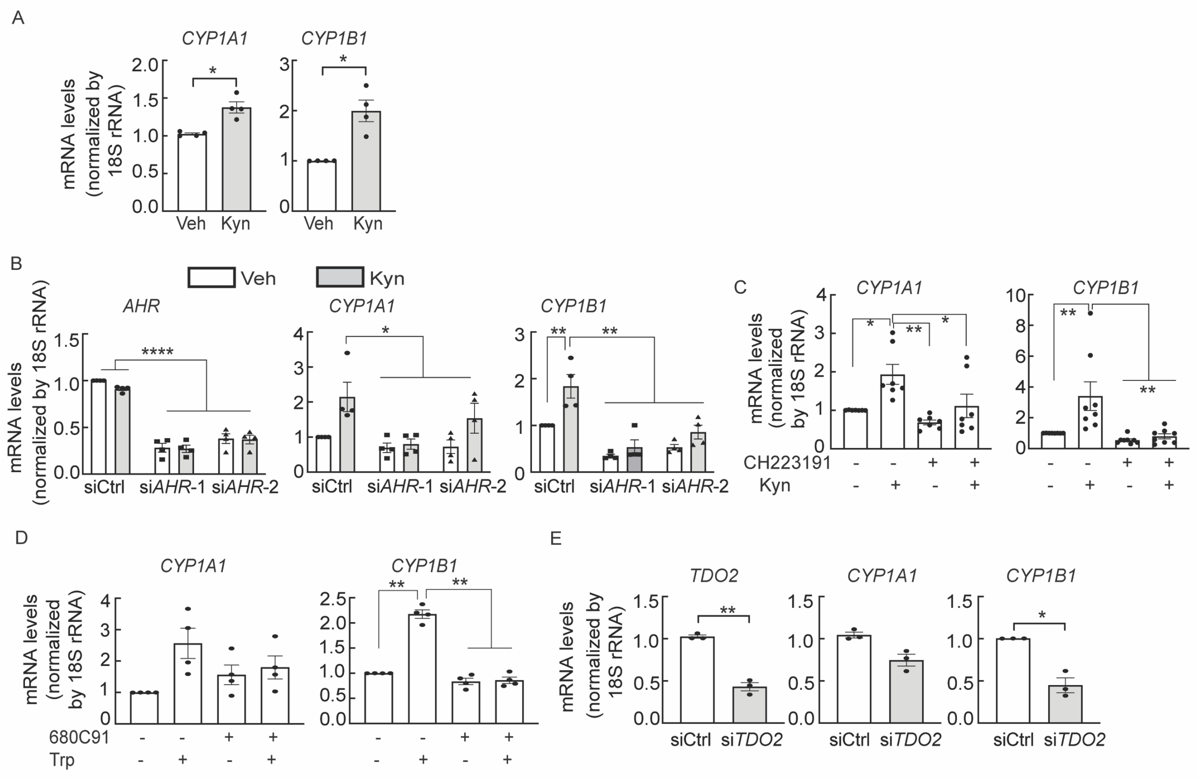

Supplemental Figure 5: TDO2-mediated Trp metabolism activates the AHR pathway in LM cells. (**A**) RT-qPCR of mRNA levels of *CYP1A1* and *CYP1B1* in LM cells treated with Kyn (200 μM) for 24 h (n=4). (**B** and **C**) Effects of *AHR* siRNA (**B**; n=4) or AHR antagonist (**C**; CH223191, 10 μM, n=7-8) on Kyn-induced *CYP1A1* and *CYP1B1* gene expression. Ctrl: control. si: siRNA. (**D** and **E**) Effects of TDO2 inhibitor (**D**; 680C91, 10 μM, n=4) or *TDO2* siRNA (**E**; n=3) on Trp-induced *CYP1A1* and *CYP1B1* expression. The mRNA levels of *AHR, TDO2, CYP1A1,* and *CYP1B1* were normalized by *18S rRNA*. *p<0.05, **p<0.01, ****p<0.0001 by Paired *t* test (A and E) or One-way (C and D) or Two-way ANOVA (B) with multiple comparison test.

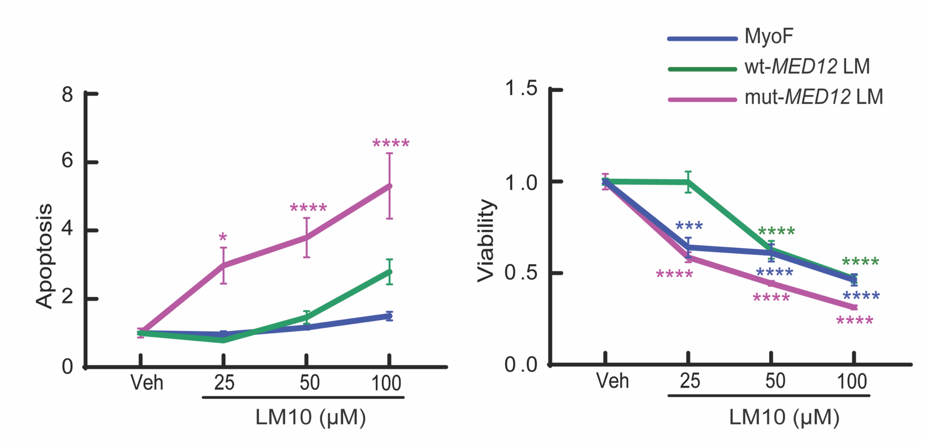

Supplemental Figure 6: TDO2-specific inhibitor LM10 inhibits cell growth more significantly in mut-*MED12* LM vs. wt-*MED12* LM. MyoF, wt-*MED12* LM, and mut-*MED12* LM cells were treated with different doses of LM10 for 48 h and analyzed for apoptosis and viability (n=5 for each tissue type). *p<0.05, *** <0.005, ****p<0.0001 by Two-way ANOVA with multiple comparison test.

Supplemental Table 1: Information about the tissue samples used in this study.

Serial No	Sample Patient record number	Age (yrs.)	Race/ Ethnicity	Tissue	Mutation	Experiments used	Smoking	No of pregnancies
1	T65	45	European American	leiomyoma, myometrium	MED12 (G44R)	WB, qRT-PCR	No	N/A
2	T66	41	European American	leiomyoma, myometrium	MED12 (G44D)	WB, qRT-PCR	No	N/A
3	T71	46	African American	leiomyoma, myometrium	MED12 (G44R)	WB, qRT-PCR	No	N/A
4	T73	41	African American	leiomyoma, myometrium	WT	WB, qRT-PCR	No	N/A
5	T74	39	African American	leiomyoma, myometrium	MED12 (Q43P)	WB, qRT-PCR	No	N/A
6	T75	36	African American	leiomyoma, myometrium	MED12 (G44D)	WB, qRT-PCR	No	N/A
7	T94	42	African American	leiomyoma, myometrium	MED12 (G44D)	WB, qRT-PCR	No	N/A
8	T115	48	European American	leiomyoma, myometrium	MED12 (G44R)	WB, qRT-PCR	No	N/A
9	T116	39	European American	leiomyoma, myometrium	WT	WB, qRT-PCR	No	N/A
10	T117	39	African American	leiomyoma, myometrium	MED12 (G44D)	WB, qRT-PCR	No	N/A
11	T126	45	Asian	leiomyoma, myometrium	WT	WB, qRT-PCR	No	N/A
12	T127	33	European American	leiomyoma, myometrium	MED12 deletion	WB, qRT-PCR	No	N/A
13	2	41	African American	myometrium	N/A	qRT-PCR	N/A	N/A
14	8	40	African American	myometrium	N/A	qRT-PCR	N/A	N/A
15	10	50	Hispanic	leiomyoma, myometrium	MED12 (G44A)	siRNA, Viability, Apoptosis	N/A	N/A
16	11	32	European American	myometrium	N/A	qRT-PCR	N/A	N/A
17	25	46	African American	myometrium	N/A	qRT-PCR	N/A	N/A
18	27	28	African American	myometrium	N/A	qRT-PCR	N/A	N/A
19	48	34	European American	leiomyoma	MED12 (G44S)	qRT-PCR	No	0
20	92	52	European American	leiomyoma, myometrium	MED12 (G44D)	Viability, Apoptosis	No	3
21	99	41	African American	leiomyoma, myometrium	MED12 (G44V)	Viability, Apoptosis, qRT-PCR	No	0
22	100	41	European American	myometrium	WT	qRT-PCR	No	0
23	118	41	African American	leiomyoma, myometrium	WT	WB, qRT-PCR	No	4
24	119	48	European American	leiomyoma	WT	Apoptosis	No	0
25	719	46	European American	leiomyoma, myometrium	MED12 (G44C)	siRNA, Apoptosis, Viability	No	2

26	721	46	European American	leiomyoma, myometrium	MED12 (G44R)	siRNA, Apoptosis, Viability	No	0
27	858	48	African American	leiomyoma, myometrium	WT	HPLC-MS/MS, RNA-seq, ChIP-seq	Former smoker	3
28	865	50	Hispanic	leiomyoma, myometrium	WT	HPLC-MS/MS, RNA-seq, ChIP-seq	No	0
29	894	44	African American	leiomyoma, myometrium	MED12 (G44R)	HPLC-MS/MS	N/A	N/A
30	903	45	Asian	leiomyoma, myometrium	MED12 (G44C)	HPLC-MS/MS, WB	No	1
31	910	51	African American	leiomyoma, myometrium	MED12 (G44D)	HPLC-MS/MS, RNA-seq, ChIP-seq	No	0
32	951	49	European American	leiomyoma, myometrium	WT	HPLC-MS/MS	No	2
33	955	41	European American	leiomyoma, myometrium	MED12 (G44D)	HPLC-MS/MS, WB	No	0
34	959	50	African American	leiomyoma, myometrium	MED12 (G44V)	HPLC-MS/MS	Former>30 days ago	N/A
35	965	40	unknown	leiomyoma, myometrium	MED12 (G44D)	HPLC-MS/MS, RNA-seq, ChIP-seq	No	0
36	967	43	unknown	leiomyoma, myometrium	MED12 (G44D)	HPLC-MS/MS, RNA-seq, ChIP-seq	No	4
37	975	40	European American	leiomyoma, myometrium	WT	RNA-seq, ChIP-seq	No	0
38	1007	48	African American	leiomyoma, myometrium	WT	HPLC-MS/MS, WB, RNA-seq	No	5
39	1008	47	African American	leiomyoma, myometrium	MED12 (G44D)	HPLC-MS/MS, WB, RNA-seq	One pack/day	1
40	1013	46	unknown	leiomyoma, myometrium	MED12 (G44S)	HPLC-MS/MS	No	N/A
41	1019	43	African American	leiomyoma, myometrium	MED12 (G44D)	HPLC-MS/MS, WB	No	3
42	1021	43	unknown	leiomyoma, myometrium	MED12 (G44D)	HPLC-MS/MS	No	4
43	1024	46	African American	leiomyoma, myometrium	MED12 (G44V)	HPLC-MS/MS	Quit in 2007	3
44	1038	49	European American	leiomyoma, myometrium	MED12 (G44D)	HPLC-MS/MS	3-4/day	0
45	1041	49	European American	leiomyoma, myometrium	WT	HPLC-MS/MS	No	0
46	1048	45	European American	leiomyoma, myometrium	WT	WB	No	2
47	1056	48	European American	leiomyoma, myometrium	WT	HPLC-MS/MS, WB	No	0
48	1109	45	European American	leiomyoma, myometrium	MED12 (IVS 1-8 T>A)	HPLC-MS/MS, WB	No	2
49	1123	48	unknown	leiomyoma, myometrium	MED12 (G44D)	HPLC-MS/MS, WB, RNA-seq	No	1
50	1147	46	European American	leiomyoma, myometrium	MED12 insertion	HPLC-MS/MS	No	2
51	1148	48	unknown	leiomyoma, myometrium	WT	HPLC-MS/MS	Former smoker	4
52	1172	41	European American	leiomyoma, myometrium	WT	Viability	No	1

53	1174	47	European American	leiomyoma, myometrium	MED12 (G44D)	HPLC-MS/MS	No	0
54	1220	48	African American	leiomyoma	WT	qRT-PCR, WB	No	4
55	1221	43	European American	leiomyoma, myometrium	MED12 (IVS 1-8 T>A)	Apoptosis, Viability, HPLC-MS/MS, WB	Former smoker	N/A
56	1237	47	African American	leiomyoma	MED12 (G44D)	Apoptosis	No	2
57	1238	44	European American	leiomyoma	WT	Viability, Apoptosis, WB	No	1
58	1244	48	Asian	leiomyoma, myometrium	N/A	Viability	No	4
59	1252	49	African American	leiomyoma, myometrium	MED12 (G44D)	HPLC-MS/MS, WB, RNA-seq	Former smoker	3
60	1255	48	African American	leiomyoma, myometrium	WT	HPLC-MS/MS	No	2
61	1256	35	Asian	leiomyoma	MED12 (G44A)	WB	No	0
62	1257	37	African American	leiomyoma	MED12 (Deletion)	Apoptosis	Occasionally	1
63	1260	46	European American	leiomyoma, myometrium	MED12 (G44R)	HPLC-MS/MS, WB	No	N/A
64	1267	48	African American	leiomyoma, myometrium	MED12 (G44D)	WB	No	4
65	1271	39	European American	leiomyoma	WT	WB	No	0
66	1274	39	African American	leiomyoma	MED12 (G44D)	Apoptosis	Marijuana	N/A
67	1281	54	African American	leiomyoma, myometrium	MED12 (G44D)	HPLC-MS/MS, WB	No	2
68	1286	37	European American	leiomyoma	MED12 (G44D)	Apoptosis	No	0
69	1287	45	European American	leiomyoma	WT	ChIP-RT, Viability, Apoptosis	No	2
70	1290	43	European American	leiomyoma	MED12 (G44D)	qRT-PCR, siRNA, Apoptosis	No	0
71	1291	32	Unknown	leiomyoma	MED12 (G44V)	siRNA, qRT-PCR, Apoptosis	No	N/A
72	1296	48	African American	leiomyoma, myometrium	MED12 (G44A)	qRT-PCR, Viability, HPLC-MS/MS	No	N/A
73	1298	47	African American	leiomyoma	WT	Viability	No	N/A
74	1305	39	European American	leiomyoma	WT	qRT-PCR, siRNA, Apoptosis	No	0
75	1307	47	European American	leiomyoma	WT	qRT-PCR	No	N/A
76	1316	42	Asian	leiomyoma, myometrium	N/A	Viability, WB, Apoptosis	No	0
77	1323	29	European American	leiomyoma	WT	ChIP-RT, Apoptosis	No	N/A
78	1331	43	Unknown	leiomyoma, myometrium	N/A	Viability	Former	N/A
79	1336	42	African American	leiomyoma	WT	siRNA	No	0

80	1342	40	African American	leiomyoma	WT	ChIP-RT, qRT-PCR, Apoptosis	No	1
81	1345	43	Unknown	leiomyoma, myometrium	MED12 (G44D)	AHR Localization, siRNA, HPLC-MS/MS	No	N/A
82	1350	44	African American	leiomyoma	Q43P	AHR Localization	No	0
83	1351	45	African American	leiomyoma, myometrium	MED12 (G44C)	qRT-PCR, Apoptosis, HPLC-MS/MS, Viability	No	4
84	1361	38	European American	leiomyoma, myometrium	MED12 (G44V)	WB, Apoptosis	No	N/A
85	1365	41	Asian	leiomyoma	MED12 (G44R)	qRT-PCR, Apoptosis	No	N/A
86	1366	50	African American	leiomyoma	MED12 (G44V)	Apoptosis	No	N/A
87	1372	50	African American	leiomyoma, myometrium	WT	Apoptosis	No	2
88	1377	36	European American	leiomyoma, myometrium	WT	Viability, Apoptosis	No	N/A
89	1388	41	European American	leiomyoma, myometrium	MED12 (G44D)	ChIP-RT, Viability	No	N/A
90	1390	40	European American	normal myometrium	N/A	Viability	No	N/A
91	1391	45	African American	leiomyoma, myometrium	MED12 (G44D)	ChIP-RT, Viability	Former smoker	N/A
92	1392	42	African American	leiomyoma, myometrium	WT	Apoptosis, Viability	Former smoker	2
93	1400	43	African American	leiomyoma, myometrium	MED12 (G44D)	qRT-PCR	Yes	N/A
94	1428	46	Unknown	leiomyoma	WT	qRT-PCR, WB	No	1
95	1432	42	Unknown	leiomyoma	WT	qRT-PCR	No	3
96	1470	44	Unknown	leiomyoma, myometrium	WT	WB, qRT-PCR	No	3
97	1482	42	Unknown	leiomyoma, myometrium	WT	WB, qRT-PCR	No	2
98	1488	49	European American	leiomyoma, myometrium	WT	WB, qRT-PCR	No	3
99	1490	53	Unknown	leiomyoma, myometrium	WT	WB, qRT-PCR	No	0
100	1506	48	African American	Leiomyoma	WT	qRT-PCR	No	1
101	1525	51	European American	Leiomyoma	MED12 (G44D)	qRT-PCR	Yes	N/A
102	1528	35	African American	Leiomyoma	MED12 (G44V)	qRT-PCR	No	2
103	1533	54	African American	leiomyoma	MED12 (G44D)	qRT-PCR	No	5
104	1548	56	Asian	leiomyoma	WT	qRT-PCR	No	1
105	20652	N/A	Unknown	leiomyoma	WT	qRT-PCR, WB	N/A	N/A
106	455213	N/A	Unknown	leiomyoma	MED12 (G44D)	WB, qRT-PCR	N/A	N/A

Supplemental Table 19

Primers

**Gene name**	**Company**	**Assay ID**	**Product name**
TBP	Thermo Fisher Scientific	4331182	TaqMan real-time PCR primer
GAPDH	Integrated DNA Technologies	Hs.PT.39a.22214836	PrimeTime Standard qPCR Assay
AHR	Integrated DNA Technologies	Hs.PT.56a.38998805	PrimeTime Standard qPCR Assay
TDO2	Integrated DNA Technologies	Hs.PT.58.3092178	PrimeTime Standard qPCR Assay
CYP1A1	Integrated DNA Technologies	Hs.PT.58.219047	PrimeTime Standard qPCR Assay
CYP1B1	Integrated DNA Technologies	Hs.PT.58.25328727.g	PrimeTime Standard qPCR Assay
IDO1	Integrated DNA Technologies	Hs.PT.58.924731	PrimeTime Standard qPCR Assay
IDO2	Integrated DNA Technologies	Hs.PT.58.3013208	PrimeTime Standard qPCR Assay
18S rRNA	Integrated DNA Technologies	Hs.PT.39a.22214856.g	PrimeTime Standard qPCR Assay

**Gene name**	**Company**	**Sequence 5'--3'**	**Usage**
CYP1A1	Integrated DNA Technologies	CCGCCACCCTTCGACA-Forward<br>CAGGCGTTGCGTGAGA-Reverse	ChIP-qPCR
CYP1B1	Integrated DNA Technologies	TGTCAGGTGCCGTGAGAA-Foward<br>CGAACTTTATCGGGTTGAA-Reverse	ChIP-qPCR

siRNAs and antibodies

**Name**	**Company**	**Cat. Number**
ON-TARGETplus Non-targeting Pool	Dharmacon	D-001810-10-05
ON-TARGETplus Human *TDO2* siRNA - SMARTpool	Dharmacon	L-008506-01-0005
ON-TARGETplus Human *AHR* siRNA - Individual	Dharmacon	J-004990-05-0005
ON-TARGETplus Human *AHR* siRNA - Individual	Dharmacon	J-004990-06-0005
Donkey anti-Rabbit IgG (H+L) Highly Cross-Adsorbed	Thermofisher scientific	A32754

AHR, RPT1 (for ICC)	Genetex	GTX22770
AHR (for ChIP)	Enzo life sciences	BML-SA210-0100
Cyclin D1	Cell signalling	2922S
TDO2	Proteintech	15880-1-AP
HRP-conjugated beta actin	Proteintech	HRP-66009

Additional references

1. Makinen N, Mehine M, Tolvanen J, Kaasinen E, Li Y, Lehtonen HJ, et al. MED12, the mediator complex subunit 12 gene, is mutated at high frequency in uterine leiomyomas. *Science.* 2011;334(6053):252-5.
2. Kampjarvi K, Park MJ, Mehine M, Kim NH, Clark AD, Butzow R, et al. Mutations in Exon 1 highlight the role of MED12 in uterine leiomyomas. *Hum Mutat.* 2014;35(9):1136-41.
3. Taylor RT, Wang F, Hsu EL, and Hankinson O. Roles of coactivator proteins in dioxin induction of CYP1A1 and CYP1B1 in human breast cancer cells. *Toxicol Sci.* 2009;107(1):1-8.
4. Buyukcelebi K, Chen X, Abdula F, Elkafas H, Duval AJ, Ozturk H, et al. Engineered MED12 mutations drive leiomyoma-like transcriptional and metabolic programs by altering the 3D genome compartmentalization. *Nat Commun.* 2023;14(1):4057.

Full unedited gel for Figure 3D

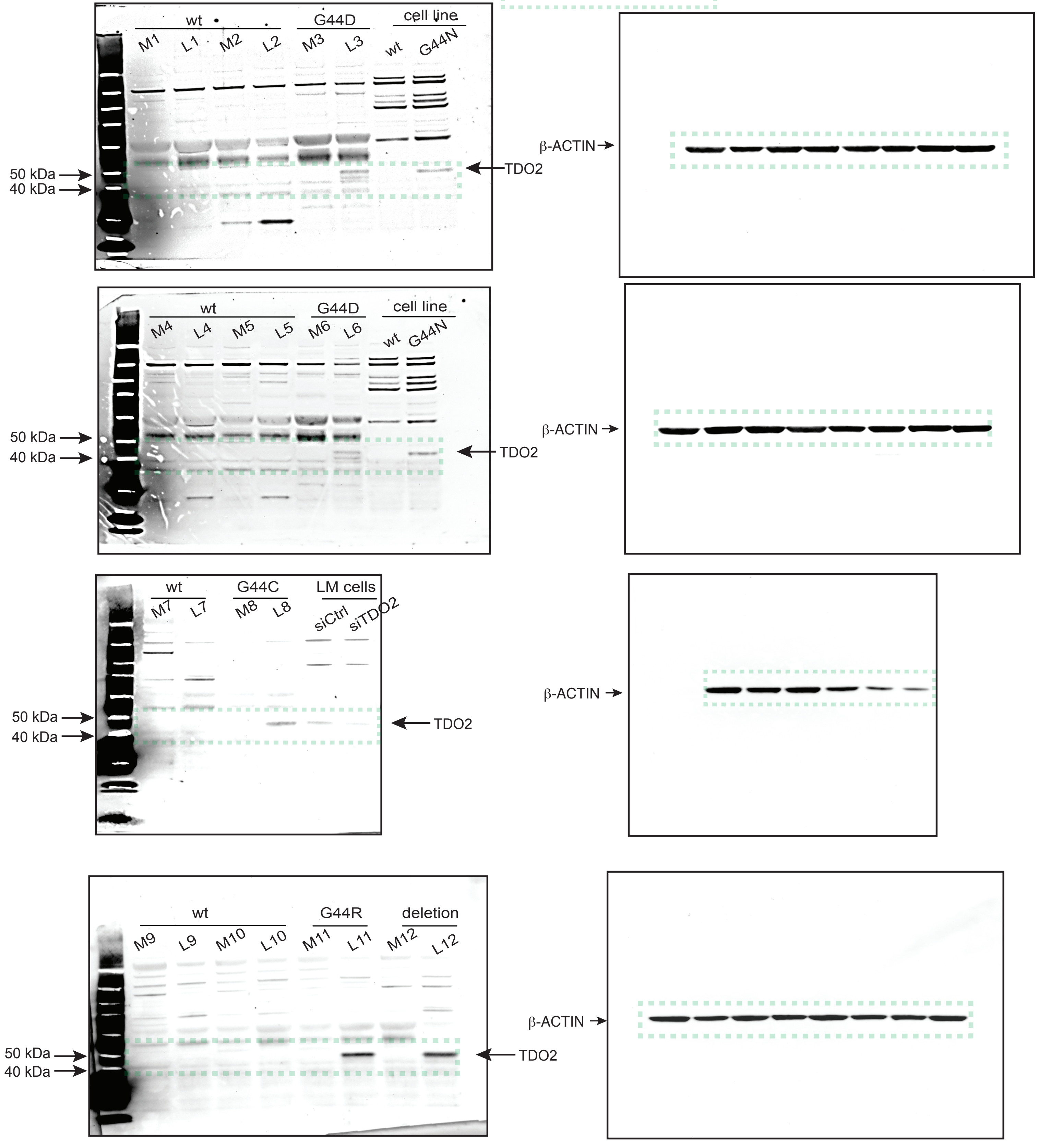

Full unedited gel for Supplemental Figure 3C

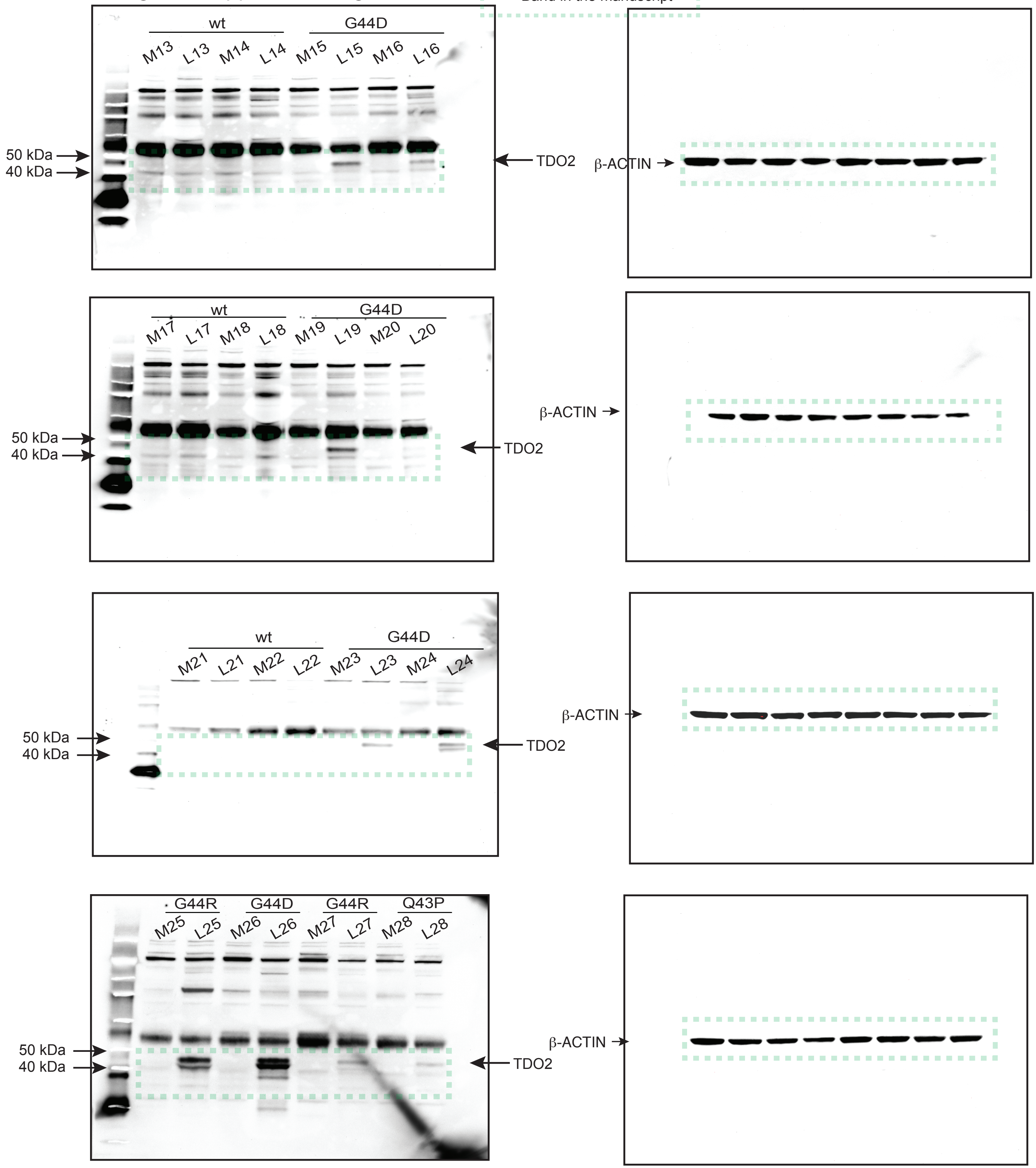

Full unedited gel for Figure 4C

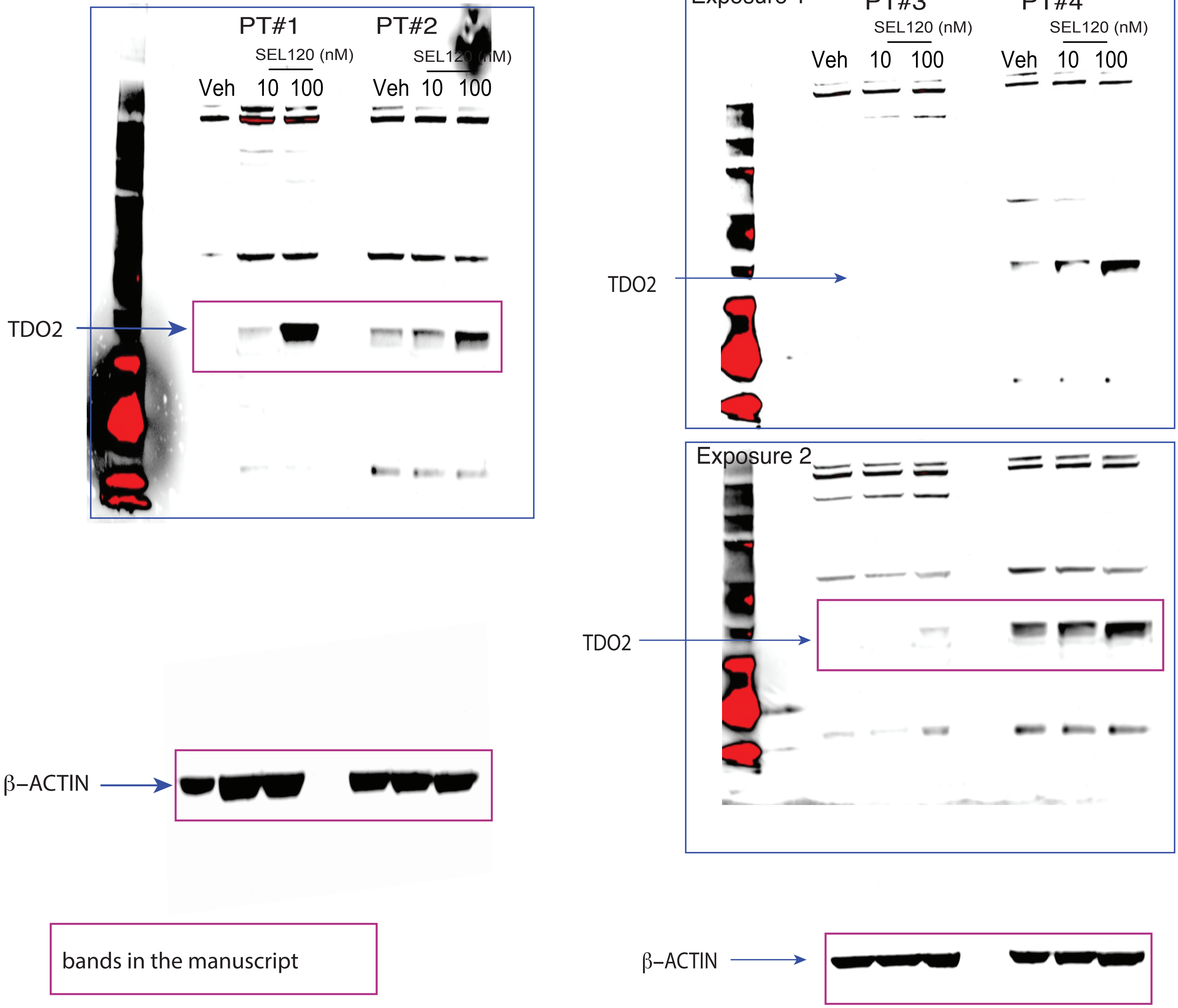

Full unedited gel for Figure 6C

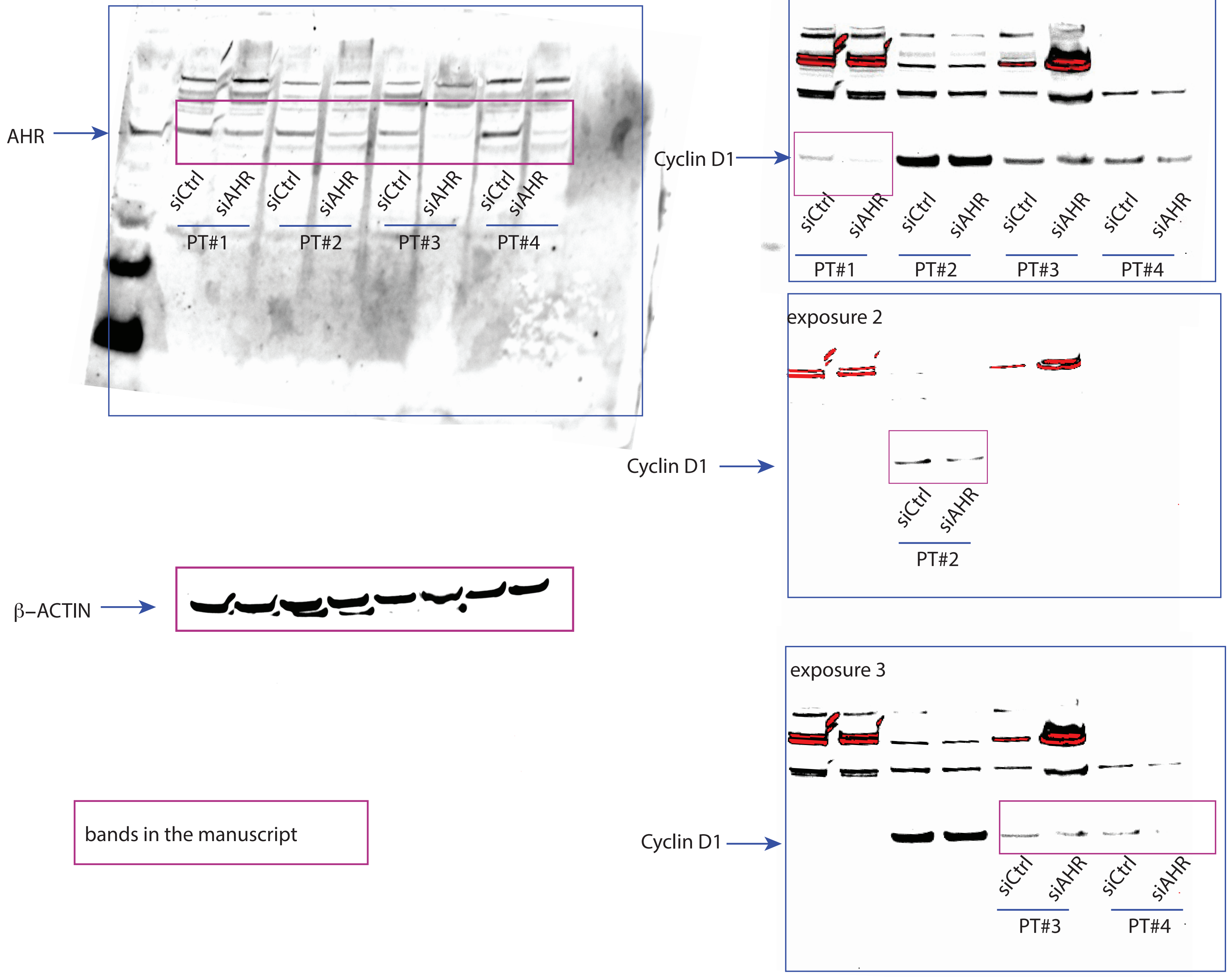

Full unedited gel for Figure 6F

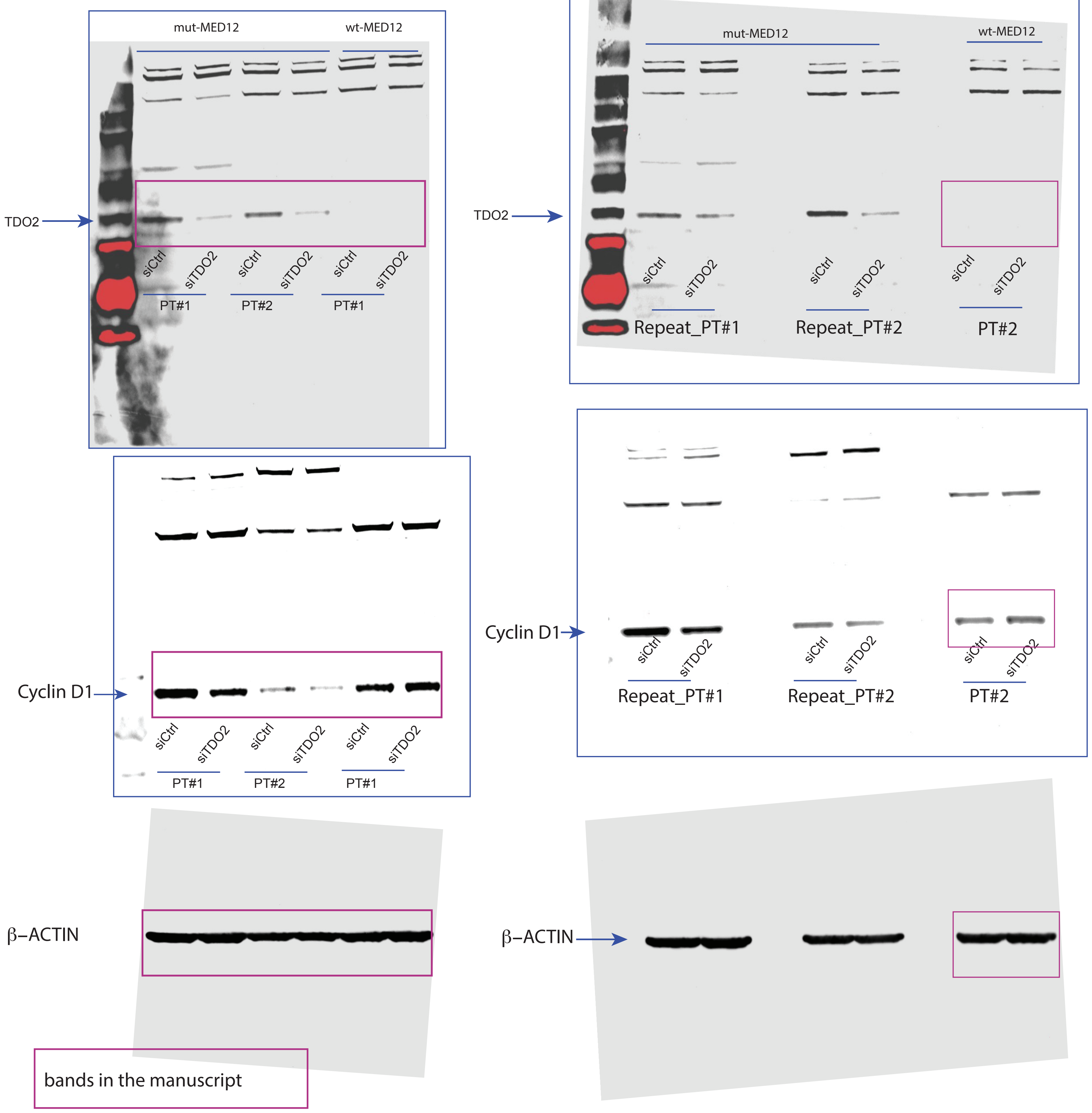

Full unedited gel for Figure 6F

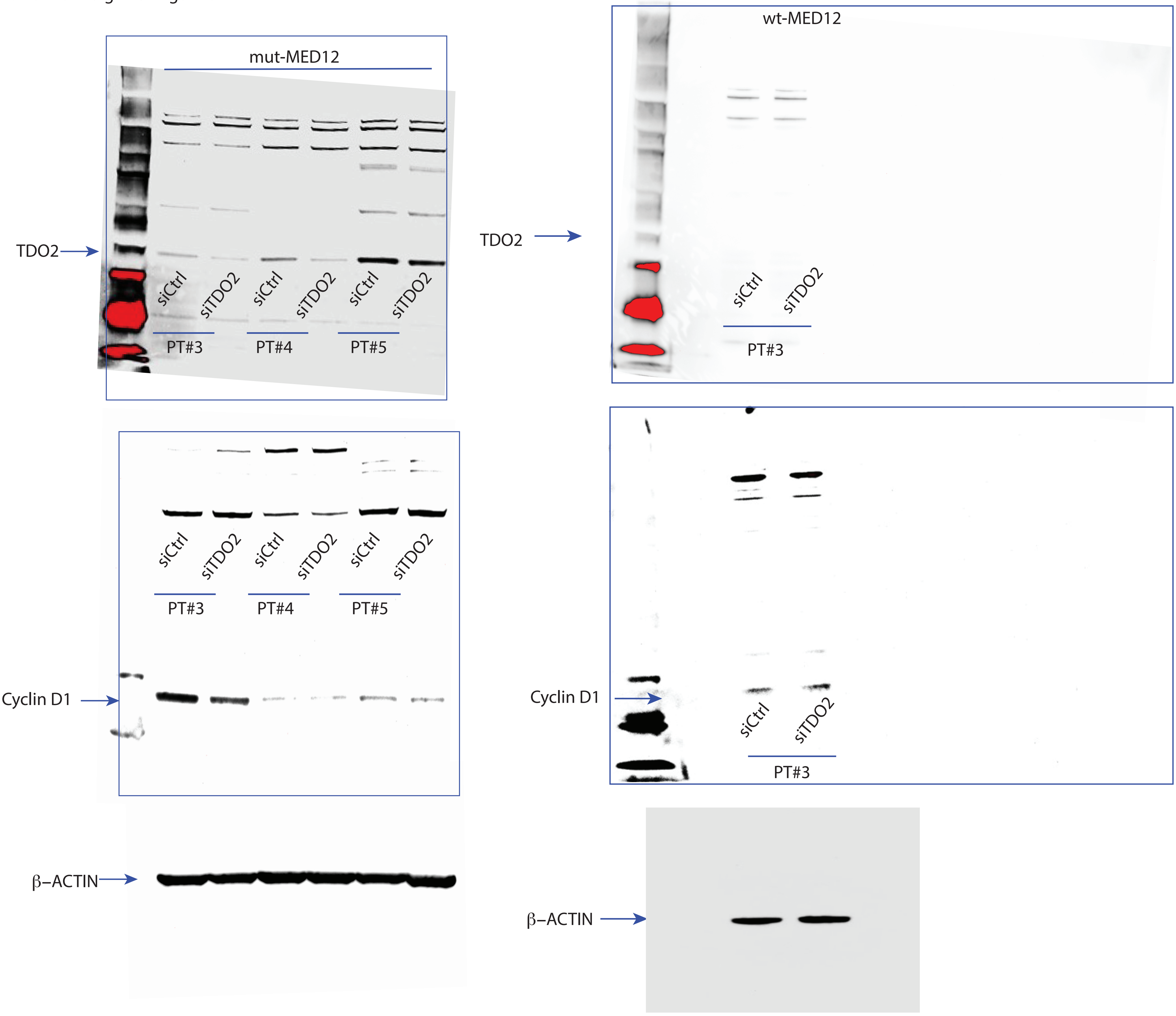

Supplement: Supplemental data [file jciinsight-8-171305-s085.pdf]
